# Dissecting serum polyclonal antibody escape to SARS-CoV-2 variants by deep mutational learning

**DOI:** 10.1016/j.crmeth.2026.101417

**Published:** 2026-04-23

**Authors:** Danielle Shlesinger, Viktor Sadilek, Mason Minot, Evangelos Stamkopoulos, Thomas Bikias, Raphael Kuhn, Andreas Agrafiotis, Joseph M. Taft, Alexander Yermanos, Sai.T. Reddy

**Affiliations:** 1 Department of Biosystems Science and Engineering, ETH Zurich, Basel, Switzerland; 2 Center for Translational Immunology, University Medical Center Utrecht, Utrecht, the Netherlands; 3 Botnar Institute of Immune Engineering, Basel, Switzerland

**Keywords:** machine learning, yeast display, immune escape, serum escape, sars-cov-2, deep sequencing

## Abstract

Deep mutational scanning (DMS) has been extensively used to investigate how single-position mutations in the receptor-binding domain (RBD) affect binding of ACE2 and neutralizing antibodies, thus revealing mutations that drive immune escape. However, these mutations represent only a small fraction of the combinatorial mutational space. Here, we present deep mutational learning (DML), a method integrating combinatorial mutagenesis, yeast surface display, deep sequencing, and machine learning (ML), to analyze serum polyclonal antibody escape. Sera from coronavirus disease 2019 (COVID-19)-vaccinated individuals were screened against diverse RBD variant libraries, validating 300 serum-variant interactions across 10 individuals. Performance remained consistent across ML architectures, suggesting that serum binding and escape are governed by distinct, localized RBD sequence features. Notably, escape profiles were highly individualized, whereas binding signatures were more conserved, reflecting convergent epitope targeting. Thus, DML can be used to generalize beyond observed RBD variants, assess cohort-specific immune breadth, and inform vaccine design in the face of viral evolution.

## Highlights

• DML maps SARS-CoV-2 serum escape across combinatorial mutational landscapes
• Ensemble models predict serum-binding phenotypes using minimally enriched yeast display data
• Serum escape profiles are individualized, while binding signatures are conserved
• Model predictions are experimentally validated on unseen RBD variants

## Motivation

The continued emergence of SARS-CoV-2 variants with multiple mutations in the receptor-binding domain (RBD) threatens the effectiveness of vaccines and antibody therapeutics. While deep mutational scanning (DMS) has mapped single-mutation effects on antibody binding and immune escape, it remains limited in resolving higher-order combinatorial landscapes. More scalable approaches are needed to characterize how polyclonal serum antibodies respond to complex RBD mutation combinations across individuals. To address these limitations, we adapt deep mutational learning (DML) to model and predict polyclonal serum reactivity across the combinatorial RBD sequence space.

## Graphical abstract

**Figure:**
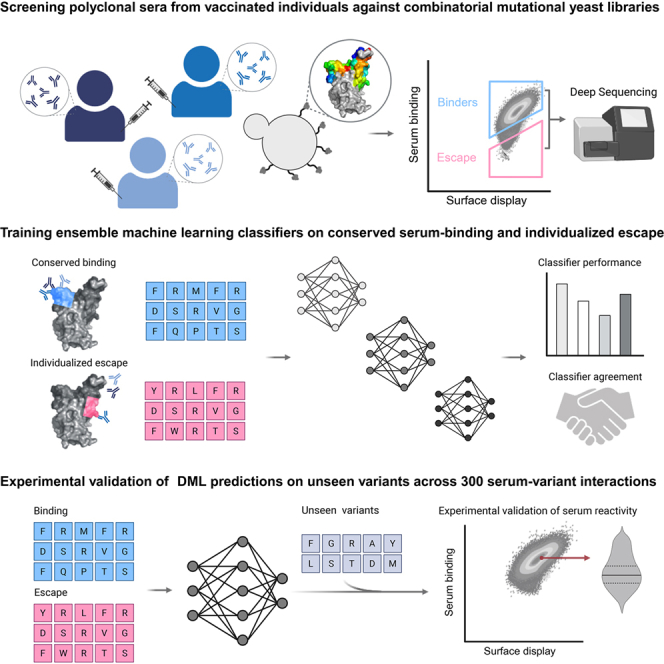

## Teaser

Shlesinger et al. show that deep mutational learning can accurately predict how human antibody mixtures recognize and escape viral variants. By combining high-throughput experiments with machine learning, the study reveals shared and individual immune patterns and provides a scalable framework to assess population-level immune coverage.

## Introduction

Since the start of the severe acute respiratory syndrome coronavirus 2 (SARS-CoV-2) pandemic, the emergence of highly transmissible and immune-evasive variants has limited vaccine efficacy and driven waves of infection, posing challenges to sustained immunity at the population level.^1^^,^^2^ The spike (S) protein is one of the structural proteins encoded in SARS-CoV-2 and forms homotrimers on the viral surface. Each monomer comprises two subunits, S1 and S2. The S1 subunit contains the receptor-binding domain (RBD), which facilitates viral attachment by specifically interacting with the angiotensin-converting enzyme 2 (ACE2) receptor, while the S2 subunit promotes viral fusion with host cells.^3^ Spanning amino acids 333–527, the RBD is the target of approximately 90% of neutralizing antibodies.^4^ Antibodies recognizing the RBD are grouped into classes: class 1–2 target the ACE2-overlapping receptor binding motif (RBM) core. Class 3 targets more conserved epitopes distal to the ACE2 site, and class 4 targets a conserved cryptic region. Vaccination tends to enrich ACE2-overlapping (class 1–2) specificities that are more escape-prone, whereas classes 3–4 can contribute broader reactivity.^5^^,^^6^^,^^7^^,^^8^ Mutations in the RBD can lead to an increase in binding affinity to ACE2 and also reduce the effectiveness of neutralizing antibodies, thereby facilitating immune escape.^9^ Traditional methods for studying antibody responses, such as serum/plasma titers measured by enzyme-linked immunosorbent assays (ELISA), are limited in throughput with regard to the number of samples and variants screened. Given the importance and abundance of mutations in the RBD that contribute to escape from neutralizing antibodies, more advanced, multidimensional approaches are needed to analyze how the mutational sequence landscape correlates with immune evasion.^10^^,^^11^^,^^12^

The protein engineering method of deep mutational scanning (DMS), coupled with yeast surface display screening, has been used extensively to investigate how single-position mutations in the RBD affect binding to ACE2 and neutralizing antibodies, thus revealing residues and mutations that drive immune escape.^10^^,^^12^^,^^13^^,^^14^^,^^15^^,^^16^^,^^17^^,^^18^^,^^19^^,^^20^^,^^21^ Additionally, screening DMS libraries against human plasma has facilitated comprehensive mapping of RBD mutations that reduce binding to polyclonal antibodies.^12^ While DMS can yield variants containing multiple mutations, these variants arise stochastically and represent only a small fraction of the combinatorial mutational space.

The emergence of Omicron and its widely circulating sublineages, with more than 15 RBD mutations compared to the ancestral SARS-CoV-2 (Wu-Hu-1), has highlighted the need to assess combinatorial mutations; however, the vast combinatorial mutational sequence space of the RBD exceeds current experimental capabilities.^10^^,^^22^ To address these limitations, we have established the method of deep mutational learning (DML), which consists of screening combinatorial mutagenesis libraries of the RBD for binding or escape to ACE2 and neutralizing monoclonal antibodies (mAbs), followed by deep sequencing. These data are used to train supervised machine learning (ML) classification models that can accurately predict whether an RBD variant binds or escapes ACE2 or neutralizing antibodies based on the sequence of the RBD.^22^ DML has also been used to determine the breadth of antibodies, allowing for the selection of neutralizing mAb combinations that optimally cover the RBD mutational sequence space, and to investigate adaptive mutational trajectories by synthetic co-evolution.^23^^,^^24^

The application of DML to serum polyclonal antibodies offers an unexplored but promising approach to study immune evasion driven by combinatorial mutations, particularly in profiling how existing or synthetic (hypothetical) viral variants escape polyclonal immune responses. Here, we apply DML to screen human serum antibody reactivity from individuals post- coronavirus disease 2019 (COVID-19) vaccination against combinatorial yeast-displayed SARS-CoV-2 RBD libraries, integrating deep sequencing and ML to predict variant binding and immune escape. Our ML models accurately predicted RBD escape mutants, were validated experimentally, and demonstrated generalizability to unseen sequences, while also revealing how the experimental screening workflow and ML model architectures critically influence predictive outcomes. By determining serum polyclonal antibody escape fingerprints, this approach advances variant risk assessment and can serve as an effective immune-monitoring tool to discern and evaluate the protective breadth of current and future vaccines.

## Results

### Serum profiling of SARS-CoV-2 RBD variants reveals patterns of binding and immune escape across individuals

**Figure 1:**
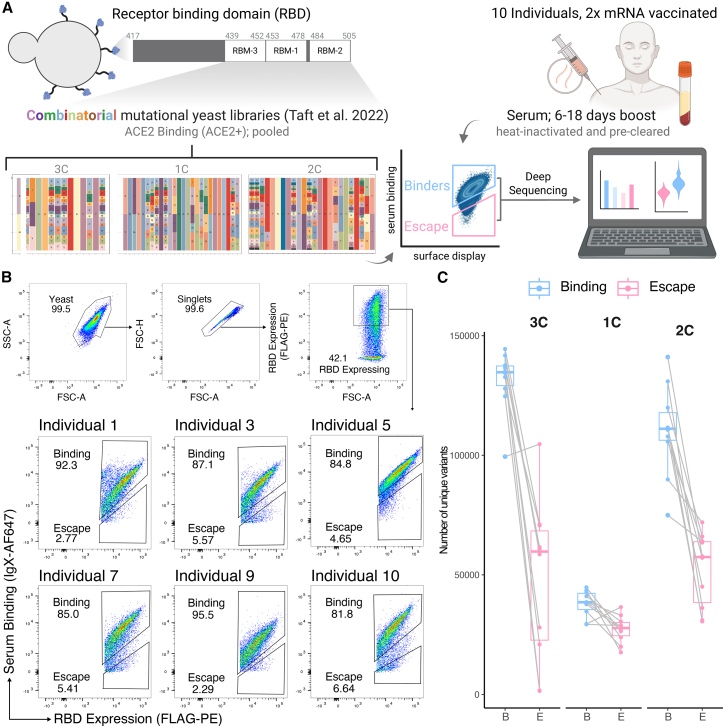
Overview of screening polyclonal serum against combinatorial yeast display libraries of SARS-CoV-2 RBD variants (A) Workflow illustrating screening of polyclonal serum followed by deep sequencing and bioinformatic analysis. A yeast surface display library with combinatorial mutations designed in the RBM of the RBD was screened against heat-inactivated, pre-cleared serum by FACS. Binding and escape populations were isolated and subsequently deep sequenced. Binding separability was evaluated and validated to ensure suitability for downstream training of machine learning models to predict serum binding and escape. (B) Example flow cytometry dot plots depicting screening of ACE2+ 1C, 2C, and 3C pooled libraries against serum. Singlets were first identified, followed by selection of RBD-expressing cells defined by expression of the Wu-Hu-1 variant, and subsequent separation into serum-binding and escape populations. (C) Paired numbers of unique serum-binding (B) or -escape (E) RBD variants retrieved from deep sequencing for each individual in each library.

DML has thus far only been applied to study SARS-CoV-2 RBD escape profiles in the context of neutralizing mAbs.^22^^,^^24^ We therefore adapted DML to predict RBD variant escape from polyclonal serum antibodies obtained from COVID-19-vaccinated individuals. To accomplish this, we leveraged three previously published SARS-CoV-2 RBD mutagenesis libraries, referred to as 1C, 2C, and 3C, which were designed using the ancestral Wu-Hu-1 sequence as a reference, and correspond to different epitope sites of the RBD, referred to as RBMs (RBM-1 [residues 452–478], RBM-2 [residues 484–505], and RBM-3 [residues 440–452]). These libraries, constructed using combinatorial mutagenesis informed by DMS data, were designed to cover a high mutational sequence space with theoretical amino acid diversities ranging from 3.5 × 10^7^–1.5×10^10^ and were previously isolated based on binding to human ACE2 receptor.^22^ To focus on biologically relevant RBD variants that show functional receptor interactions, we pooled the ACE2+ fractions of the three separately assembled 1C, 2C, and 3C libraries in equal ratios into one combined library. Notably, these libraries were previously validated, showing escape from class-defined mAbs targeting structural epitopes.^22^ We screened this library against ten serum samples obtained from individuals following two-dose vaccinations (6–18 days post-second vaccination) during the early stages of the pandemic (early 2021) (Table S1). The serum was heat-inactivated and pre-cleared with uninduced yeast cells to remove yeast-binding antibodies prior to fluorescence-activated cell sorting (FACS). Serum-binding and escape variants were isolated, and targeted deep sequencing (Illumina) of the RBD region was performed. Across vaccine types, serum-binding signal distributions were similar, and inter-donor differences (escape-gate 2.66–9.85%) aligned with previously reported heterogeneity and likely reflected repertoire variation (Figures 1A and 1B; Figures S1A, S1B, and S1C).^17^ In total, 3,037,678 binding and 1,423,537 escape sequences were retrieved, with overall fewer sequences originating from sublibrary 1C compared to 2C and 3C (Figure 1C).

**Figure 2:**
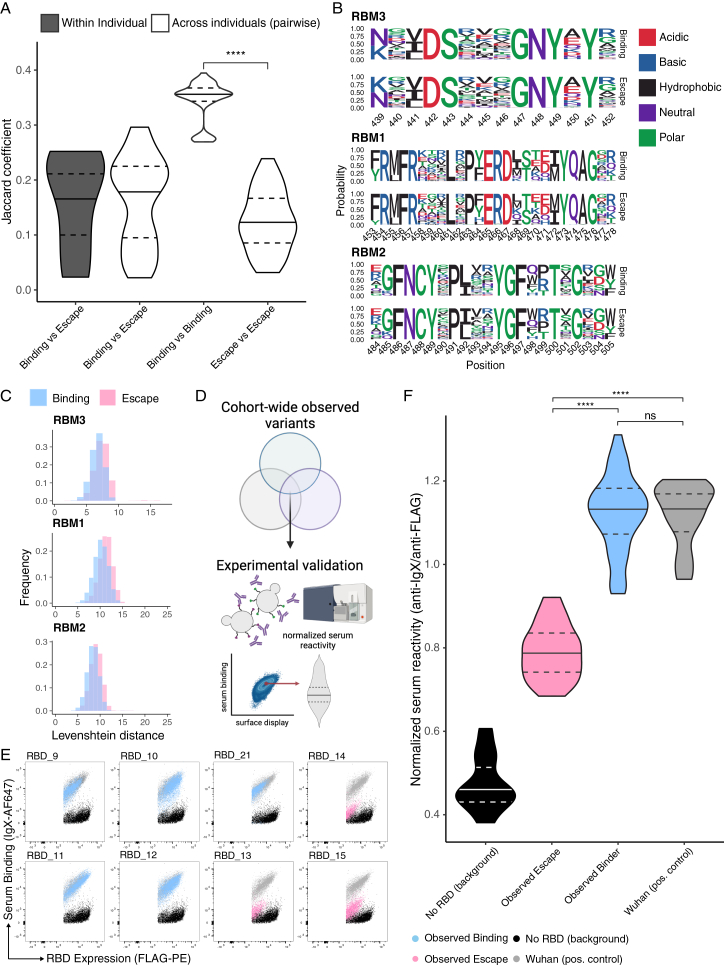
Screening of polyclonal serum against combinatorial yeast display libraries can recover separable serum-binding and -escape RBD variants with experimentally verified binding profiles (A) Violin plot depicting the Jaccard index between binding and escape fractions within and across individuals. (B) RBM protein sequence logo plots from serum-binding and escape variants. Sequences with mixed labels were removed across the cohort. (C) Comparison of distributions of mutational distances of RBMs from serum-binding and escape fractions. Mutational distance was measured as Levenshtein distance from the Wu-Hu-1 sequence. Sequences with mixed labels were removed across the cohort. (D) Overview of validation experiments for observed variants. Cohort-wide binding and escape variants were expressed individually on yeast cells and screened against all individual serum samples by flow cytometry. All variants were selected solely based on fraction identity (binding-only or escape-only across all donors); no classifier was used for selection. Normalized serum reactivity was calculated as the mean ratio of serum binding signal (anti-IgX) over RBD expression signal (anti-FLAG). (E) Representative FACS plots from screening one serum against observed RBD variants. (F) Group comparison of normalized serum reactivity (mean signal ratio anti-IgX/anti-FlAG) for observed serum-binding and escape variants. Statistical analysis was performed using an unpaired two-tailed Student’s *t* test and is shown for observed binders vs. escape variants and relative to the Wu-Hu-1 positive control. “No RBD” serves as a background reference rather than a true negative control. By design, all groups are significantly above “No RBD”; full pairwise statistics including EV comparisons are provided in Table S3. ∗*p* < 0.05, ∗∗*p* < 0.01, ∗∗∗*p* < 0.001, ∗∗∗∗*p* < 0.0001, not significant (ns).

We first examined the extent of sequence overlap between the classification labels of binding and escape within and across individuals, as the presence of overlapping RBD sequences (mixed labels) could complicate the training of downstream ML models. To this end, we calculated the Jaccard index (defined as the intersection divided by the union) within and across individuals, which demonstrated that binding and escape fractions showed varying degrees of similarity within and across individuals. Notably, serum antibody-binding RBD variants were significantly more shared across individuals compared to serum antibody-escape variants (Figure 2A). We further examined whether removing sequences with mixed labels within or across individuals would impact different similarity measures. Filtering decreased similarities overall, with binding sequences remaining more similar to each other than escape variants. Protein sequence logo plots revealed high similarity in amino acid usage between serum-binding and -escape variants, which was only minimally affected by the filtering strategy (Figures 2B, S1D, S2A, and S2B). As SARS-CoV-2 variants mutate to evade immune protection, we hypothesized that the sorted RBD escape variants would demonstrate larger mutational distances to the ancestral Wu-Hu-1 sequence compared to serum-binding sequences. We therefore calculated the Levenshtein distance in both serum-binding and -escape sequences across all RBMs. Serum-escape variants demonstrated a slightly higher Levenshtein distance from the Wu-Hu-1 sequence across all RBMs. These differences were more pronounced when mixed-label sequences were removed, highlighting that removing mixed-label sequences may improve class separability (Figures 2C, S2C, and S2D). We next contextualized escape by mapping the position-wise difference in mutation frequency (Δf; escape - binder) onto the RBD structure. This highlighted discrete hotspots, mainly in RBMs 1 and 2, which overlap ACE2-facing surfaces commonly targeted by class 1 and 2 antibodies that are known to be more escape-prone.^8^^,^^17^ Additionally calculating and mapping a compact chemical switch score, summarizing the propensity for property-altering versus property-preserving substitutions, indicated that many hotspot sites favor property-altering changes, suggesting a preference for changes in amino acid properties and, thus, a substantial alteration of the local chemical environment (Figure S2E). Given the sequence similarity of serum-binding and -escape variants and their FACS gating proximity, we next experimentally validated the binding properties of observed sequences. To this end, we selected eight RBD variants that were present exclusively in binding or escape fractions in all ten individuals, produced them as single variants by yeast surface display, and tested them for serum binding by flow cytometry (Table S2). This analysis directly confirms the fidelity of our FACS sorting strategy and the clear separation of binding phenotypes before any DML models were trained, providing confidence in our experimental labels. We calculated the normalized serum reactivity, which is defined as the mean ratio of the serum-binding signal (anti-IgX) over the RBD-expression signal (anti-FLAG) for each variant, and compared this to the mean ratio of the Wu-Hu-1. The calculated ratio reflects the normalized reactivity of the serum against the RBD, accounting for variations in RBD expression (Figure 2D). “No RBD” (yeast displaying all elements of the construct except the RBD) was included as a visually informative baseline reference, as polyclonal serum engages multiple epitopes simultaneously. Responses therefore typically exceed this background. In contrast to the observed binders, observed escape variants had significantly lower normalized serum reactivities compared to Wu-Hu-1 (Figures 2E and 2F). Directly comparing observed serum-binding and -escape variants demonstrated significantly different normalized serum reactivities, further validating the sensitivity of our yeast display and FACS workflow to produce accurate labels (Figures 2F and S2F). Although sequence similarity exists between serum-binding and -escape RBD variants, sorting combinatorial mutagenesis yeast libraries against polyclonal serum once can recover millions of RBD sequences, with serum-escape variants that exhibit higher mutational distances than binders.

### Individual-specific and cohort-wide classifiers accurately predict serum-escape variants

**Figure 3:**
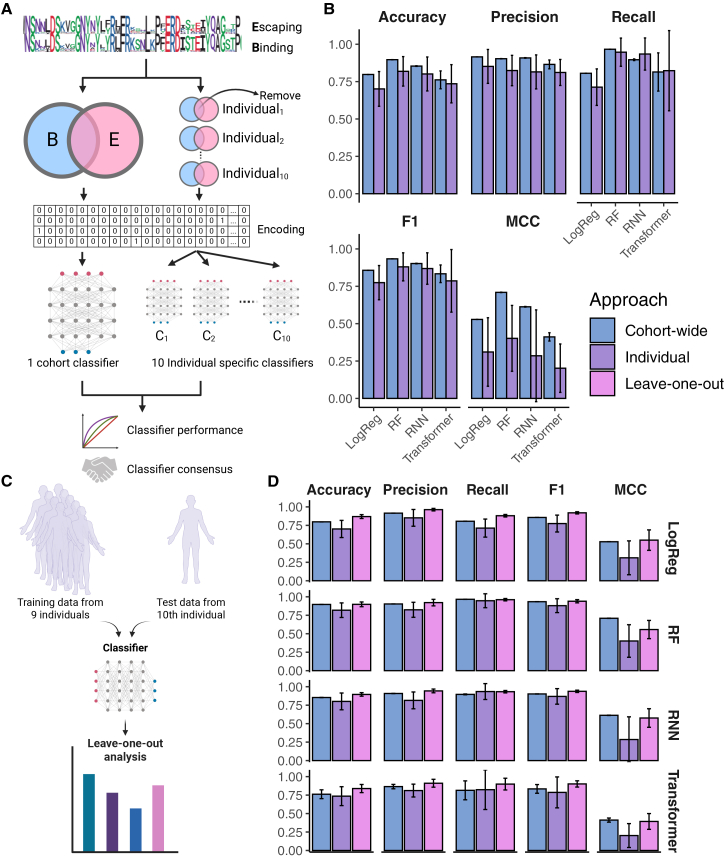
Cohort-wide machine learning models accurately predict serum antibody-binding and escape variants (A) Computational workflow for machine learning analysis of serum-screened RBD variants: recovered sequences were stratified into either cohort-level (left) or individual-specific (right) sets of serum-binding and escape variants. Variants with mixed class labels were removed from the data prior to training individual-specific or cohort-wide classifiers. Classifiers were trained to predict serum binding or escape phenotypes of an RBD based on sequence. (B) Comparison of performance metrics across averages of different individual-specific classifiers and a cohort-wide classifier, including logistic model (LM), random forest (RF), recurrent neural network (RNN), and Transformer models. (C) Computational workflow of the “leave-one-out” approach. (D) Ten RF classifiers were trained using a leave-one-out strategy, where each model excluded one individual for performance evaluation. Results are compared against individual-specific and cohort-wide classifiers. Standard deviations are shown as error bars and represent training across individuals and random-seed runs.

DML relies on using ML models to accurately predict binding and escape profiles of experimentally unscreened RBD variants, thereby enabling extrapolation over a broader mutational sequence space compared to experimental methods such as DMS.^22^^,^^24^ Mixed labels can introduce ambiguity and impair a classification task, regardless of whether a mixed label accurately represents the underlying data. Our previous results uncovered the presence of mixed labels both within and across individuals (Figure 2A). To address ambiguity in RBD variants appearing in both binding and escape groups, we employed two complementary strategies: (i) individual-specific filtering, removing sequences with conflicting labels within each donor and training 10 donor-specific classifiers to capture personalized RBD serum profiles (Figure 3A); and (ii) cohort-wide filtering, eliminating all overlapping sequences across the entire cohort to train a single consensus model. This dual approach distinguishes whether mixed labels reflect biological individual variation (supported by superior individual model performance) or technical noise (indicated by cohort model performance) (Figure 3A).

In our previous DML study, we utilized an ensemble model approach with random forests (RFs) and recurrent neural networks (RNNs).^22^ To capture both position-specific and longer-range sequence-function relationships, here we additionally included a logistic regression model for interpretable, parameter-low predictions, and protein language model (PLM) embeddings, ESM2-650M (Transformer-based), paired with a logistic regression classification head to incorporate deep contextual learning via transfer learning for parameter-rich modeling.^25^ We screened serum against the pooled 1C, 2C, and 3C sub-libraries and subsequently trained all ML models on the combined RBD sequence region (position 417–505). To reduce bias in the training data, we balanced both label and sub-library, ensuring an equal representation of serum-binding and -escape variants, as well as variants from each sub-library. In both approaches and across all tested architectures, the RF model best predicted RBD escape variants, with cohort-wide classifiers achieving F1 (harmonic mean of precision and recall) and MCC (Matthews correlation coefficient, a balanced measure of binary classification performance) scores of 0.93 and 0.71, respectively, whereas individual classifiers resulted in mean values of 0.88 and 0.40, respectively (Figure 3B). The performance of individual classifiers varied strongly across individuals and correlated with the number of sequences (amount of training data) retrieved for the corresponding individual (Figure S3A). This correlation underscores the benefit of the cohort-wide classifier, as the cost of deep sequencing escalates with increasing cohort sizes. We next assessed the ability of the cohort-wide classifier to generalize to unseen individuals and determined whether individual-specific patterns might limit its effectiveness. To this end, we performed a “leave-one-out” training and testing approach, corresponding to ten different cohort-wide classifiers (Figure 3C), each of which was trained using data from all individuals except one. To ensure there was no data leakage between training and test data, any overlapping sequences observed in a given individual and the rest of the training data were filtered out. This approach was comparable to the cohort-wide approach and resulted in a mean F1 score of 0.94 and MCC of 0.55 (Figure 3D). This implies that the distribution of serum antibody binding and escape variants learned at the cohort level is reflected at the individual-specific level. At the resolution of yeast display and the available sequencing depth, individual antibody landscapes appear broadly similar, with no detectable differences in individual escape patterns (Figure S4). Thus, cohort-level classifiers may perform as effectively as individual-specific models in predicting serum antibody binding and escape profiles.

Screening yeast libraries often requires multiple enrichment rounds to reduce noise, which can be labor-intensive, costly, and limited by sample availability.^26^ We conducted two enrichment rounds for four individuals, finding that while enrichment reduced overlap between binding and escape fractions (Jaccard indices: 0.001–0.085 within individuals, 0.001–0.082 across individuals), it also increased the difference in Levenshtein distances between serum binding and escape variants, and enhanced classifier performance (F1 score: 0.98, MCC: 0.92) (Figures S3B, S3C, and S3D). To reduce experimental effort, we created a mixed dataset by combining first-sort serum-binding variants with third-sort escape variants, achieving comparable performance (F1 score: 0.99, MCC: 0.97) to fully enriched data (Figure S3E). Our results suggest that enriching only the escape population may be sufficient to generate high-quality training data while conserving experimental resources. Given the strong performance from single-sort data, the remainder of this study focuses on these single-sort datasets to further explore these outcomes.

### Experimental validation demonstrates DML can accurately predict serum binding and escape variants

**Figure 4:**
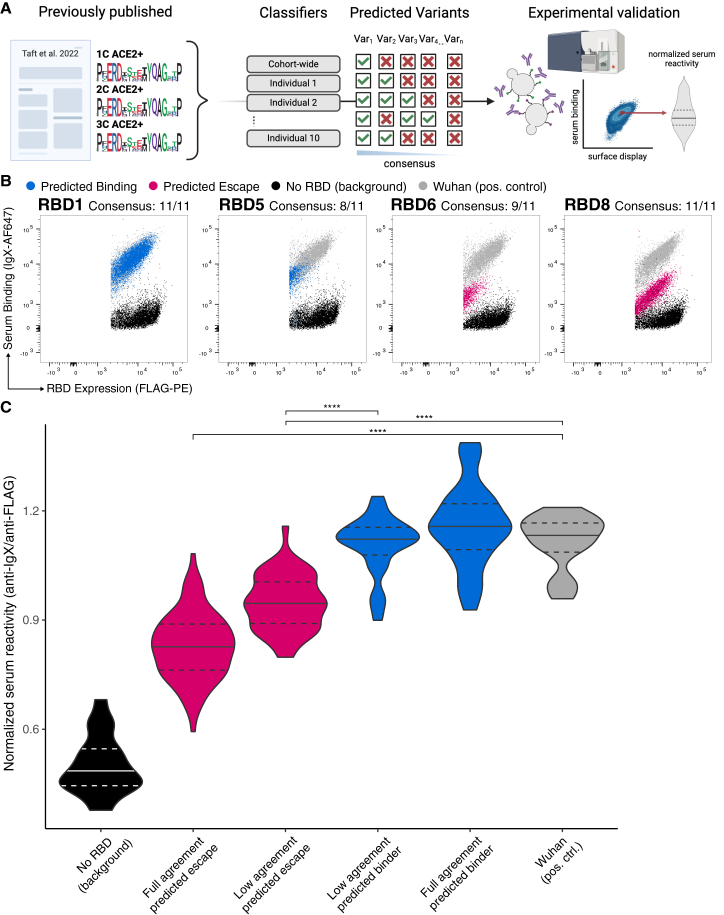
High-consensus predictions of trained classifiers are experimentally validated (A) Workflow for the experimental validation of unseen RBD variants (not present in the training or test data). (B) Representative FACS plots from screening serum against unseen RBD variants, where the ensemble of all models showed full agreement. Gating strategy is as described in Figure 1B. (C) Comparison of normalized serum reactivities (anti-IgX/anti-FlAG) for predicted serum-binding and escape variants with either full or low ensemble model agreement. The center line indicates the median and dashed lines indicate the interquartile range. Statistical analysis was performed using an unpaired two-tailed Student’s *t* test and is shown for predicted binders vs. escape variants and relative to the Wu-Hu-1 positive control. “No RBD” serves as a background reference. By design, all groups are significantly above “No RBD”; full pairwise statistics, including “No RBD” comparisons, are provided in Table S3. ∗*p* < 0.05, ∗∗*p* < 0.01, ∗∗∗*p* < 0.001, ∗∗∗∗*p* < 0.0001, not significant (ns).

After observing high accuracy of the RF classifier in predicting binding versus escape, we aimed to experimentally validate the performance of both the individual and cohort-wide classifiers on unseen RBD variants. These variants were not present in our training or test sets and were sourced from the previously published ACE2+ unpooled 1C, 2C, and 3C libraries.^22^ To validate our predictions, we expressed 15 RBD variants individually on yeast that corresponded to full prediction agreement across all eleven models (cohort-wide and ten individual classifiers) and experimentally tested them for serum binding (Figure 4A). Furthermore, we ensured variants were sampled from different sub-libraries and had varying mutational distances from Wu-Hu-1 (Table S2). Notably, across all predictions, there was generally more consensus for the prediction of binders, with 48.92% of the predicted variants classified with full consensus as binders, compared to only 0.04% as escape (Figure S5A). To analyze the serum-binding phenotypes of all produced variants across all ten individuals, we again measured the normalized serum reactivity for each variant and compared this to the mean ratio of the ancestral Wu-Hu-1 RBD. Variants predicted to escape with full consensus showed significantly lower normalized serum reactivity compared to the positive control (Wu-Hu-1), whereas full-consensus predicted binders had similar or higher normalized serum reactivity compared to the positive control. Predicted serum-binding and escape-variants substantially differed in their normalized serum reactivity, highlighting their distinguishable binding profiles (Figures 4B and 4C).

Given the lower performance of our individual classifiers compared to the cohort-wide classifiers, we next focused on assessing the binding profiles of RBD variants where predictive discrepancies between models occurred. We selected and screened seven variants where the ensemble models did not reach full agreement. The selected variants were expressed on yeast and then evaluated for serum binding, and their normalized serum reactivity was measured. Those RBDs in which the majority of models predicted binding had similar normalized serum reactivity compared to the positive control, while variants partially predicted to escape had significantly lower normalized serum reactivity (Figures 4B and 4C). This demonstrated that, when using our ensemble approach for prediction, the majority of the ensemble correctly coincides with serum binding and escape. Notably, the normalized serum reactivity of low-agreement variants was closer to each other compared to full-agreement predicted variants, indicating that the level of agreement could be associated with reduced separability of the serum reactivity (Figures 4C, S5B, and S5C).

We further investigated the models that deviated from the majority consensus. In only one of the seven produced variants did the cohort-wide classifier assign the wrong label, which also matched the minority prediction among all models (Table S2; RBD7). More detailed analysis of the normalized serum reactivity showed that low-agreement binders were correctly predicted by the majority of the ensemble, highlighting our previous finding that our models show greater agreement in identifying binders (Figures S5A and S6A). In contrast, for low-agreement escape variants, one of the two minority models correctly defied the ensemble (Figure S6A; RBD6, individual 8). Moreover, there were also cases where single individual responses did not align with the majority prediction, with single-individual serum reactivities being close to the positive control (Figure S6A). Notably, visual inspection of the FACS plots showed that many of the produced variants with low agreement had low expression profiles (Figure S6B). This suggests that disagreement among the models can reflect the uniqueness of individual antibody landscapes in rare cases and additionally points to a challenging region of the sequence space that is difficult to predict. Overall, our findings suggest that a cohort-wide classification approach is generally sufficient to accurately predict serum binding profiles, as individual serum landscapes appear functionally similar when assessed with yeast display at the given sequencing depth. However, disregarding individual labels can reduce classification accuracy, underscoring the advantage of an ensemble approach despite its higher computational cost. These results demonstrate that even with single-sort data, DML can effectively predict serum binding and escape using ensemble modeling.

## Discussion

Here, we extend the application of DML to predict serum polyclonal antibody binding and escape across SARS-CoV-2 RBD variants. While prior DMS studies have validated single-mutation escape variants by identifying their enrichment in circulating immune-evasive strains and linking them to reduced neutralization capacity, these approaches are limited in capturing the complexity of combinatorial mutations.^12^ In a previous study, we demonstrated that DML could accurately identify multi-site mutations driving escape from therapeutic mAbs, validating 46 synthetic RBD variants across four antibodies with over 90% accuracy and a total of 178 experimental validations.^22^^,^^24^ Building on this foundation, we now adapt the DML framework to analyze more complex polyclonal sera, addressing key challenges such as label noise, limited sample availability, and the inherent heterogeneity of individual serum responses in high-throughput experimental workflows.

High-throughput screening methods inherently produce noisy data, and FACS-based yeast surface display screens, commonly employed for mapping binding interactions, often require multiple rounds of enrichment to clearly distinguish binding from escape populations.^26^^,^^27^ This iterative process is both time-consuming and constrained by limited sample availability, particularly when attempting to isolate rare binding events. Although recent meta-learning approaches have shown promise in accelerating screening and enhancing model robustness, they still rely on a small, trusted meta-set, typically derived from multiple rounds of enrichment.^28^ In this study, we demonstrate that DML can accurately recover distinct binding labels using only once-sorted data by computationally filtering mixed-label populations. While additional enrichment modestly reduced label ambiguity, it had only a minor effect on overall model performance, suggesting that selective enrichment of underrepresented populations may be sufficient, with computational strategies effectively compensating for reduced experimental depth.

Our DML analysis revealed inter-individual differences in the frequency of escape variants (2.77%–9.85%), which are in line with ranges reported in previous studies across donors and replicate libraries.^12^^,^^29^ Consistent with these studies, we also found significant person-to-person variability as it relates to RBD mutations on serum escape, with discrepancies between individual responses and consensus predictions occurring predominantly for escape variants. This variation likely reflects a combination of biological differences in immune history and technical factors, including donor-specific gating to account for serum dilution, titer, binding specificity, and background signal. In contrast, serum-binding variants were more consistently predicted across individuals, likely reflecting convergent targeting of shared neutralizing and non-neutralizing epitopes.^6^^,^^10^^,^^30^^,^^31^^,^^32^^,^^33^^,^^34^^,^^35^ These findings underscore the personalized nature of escape responses and the relative predictability of binding patterns, particularly within the RBD. Within this framework, the 0.04% of unseen variants classified as escape reflects a deliberately strict all-11-model consensus suited to single-sort data. While relaxing the consensus would raise apparent escape toward the ∼4.75% experimental mean but reduce specificity, future studies using additional enrichment and larger cohorts could choose intermediate, cohort-level consensus to balance sensitivity and specificity. Recent evidence suggests that immune history shapes the specificity of serum neutralizing antibodies, implying that model generalizability may improve when stratifying individuals by shared immune backgrounds.^36^ Supporting this, we observed only minor performance differences among logistic regression, RF, RNN, and transformer models, suggesting that escape and binding features are often governed by localized, interpretable sequence patterns. In the context of polyclonal sera, however, simpler models may suffice due to the dominant influence of direct sequence features and the obscured impact of conditional or allosteric interactions present in more focused antibody systems, particularly when training with limited sequence context.

While neutralizing antibodies are essential for blocking viral entry, antigen-binding antibodies also contribute to immune defense through alternative mechanisms, such as Fc-mediated effector functions.^37^^,^^38^ Notably, certain mutations can reduce antigen binding without significantly impairing neutralization, likely due to conformational differences between isolated and native RBD structures.^12^ Tools such as the DMS-based “escape calculator” have shown correlations with neutralization assays for polyclonal sera but depend on the assumption that mAb activity sufficiently captures the complexity of polyclonal responses.^39^ Our study specifically examines vaccinated individuals, who tend to exhibit higher levels of non-neutralizing antibodies compared to those with natural infection.^32^ As the serum samples were collected shortly after two-dose vaccination, our analyses relied on libraries constructed from the ancestral Wuhan-Hu-1 sequence. Future studies should incorporate Omicron-derived libraries, including our recently reported full-RBD mutational library, to capture more recent escape pathways.^24^ Moving forward, it will be important to assess how yeast display-based serum binding assays relate to real-world protection or susceptibility to emerging SARS-CoV-2 variants. DML is not intended to predict which variants will emerge, as this is a complex problem that likely goes beyond just ACE2 binding and antibody escape and includes population- and host-level (e.g., chronic infection) factors.^40^ Instead, the DML framework provides a reusable way to quantify how combinatorial RBD mutations affect polyclonal serum binding. Moreover, DML is most informative at the cohort level, enabling comparisons of escape profiles across vaccine platforms, time points, and immune backgrounds. Aggregating across donors mitigates individual variability and can highlight broadly escaping changes for surveillance and follow-up. By integrating epidemiological and clinical datasets, DML could help relate cohort-level binding patterns to real-world outcomes. Such insights could inform the selection of broadly effective antibody therapies and contribute to the design of new vaccines. Ultimately, this approach may help identify serum-binding profiles linked to durable immunity, thereby enhancing our capacity to tailor interventions for sustained population-level protection.

### Limitations of the study

Serum samples were collected shortly after two-dose vaccination, and all analyses relied on libraries derived from the ancestral Wuhan-Hu-1 RBD, limiting direct assessment of escape pathways relevant to later variants such as Omicron. In addition, yeast surface display-based assays measure serum binding rather than neutralization or *in vivo* protection and, therefore, may not fully capture functional immunity. Finally, while DML enables systematic characterization of antibody-binding landscapes, it does not account for broader evolutionary pressures that influence viral emergence beyond antibody escape and ACE2 compatibility.

## Resource availability

### Lead contact

Further information and requests for reagents, resources, or any additional information required to reanalyze the data reported should be directed to and will be fulfilled by the lead contact, Sai T. Reddy (sai.reddy@ethz.ch).

### Materials availability

Antibody mutagenesis libraries and recombinant DNA used in this study will be made available on request to the lead contact upon completion of a materials transfer agreement.

### Data and code availability

• The main data supporting the results of this study are available within the paper and its supplemental information. Raw deep sequencing data have been deposited at the NCBI SRA (accession number: PRJNA1264489) and are publicly available as of the date of publication.
• The code is deposited at Zenodo (https://doi.org/10.5281/zenodo.18660254) and Github (https://github.com/LSSI-ETH/Serum_DML_2025.git) and is publicly available as of the date of publication.
• Any additional information required to reanalyze the data reported in this paper is available from the lead contact upon request.

## Acknowledgments

We thank Christian Beisel, Mirjam Feldkamp, Elodie Burcklen, and Ina Nissen at the 10.13039/501100003006ETH Zurich D-BSSE Genomics Facility, Basel, for their support and assistance. We additionally acknowledge and thank Di Tacchio Mariangela, Cavallini Chiara, and Gumienny Aleksandra from the D-BSSE FACS facility for their support. Funding: This work was funded in part by 10.13039/501100003006ETH Zurich, the Foundation Immune Engineering for Global Child and Adolescent Health, and the Basel Research Center for Child Health. Additionally, this work was supported by a Swiss National Science Foundation (SNSF) Ambizione grant (PZ00P3_208734) to A.Y.

## Author contributions

D.S., S.T.R., and A.Y. contributed to study design. D.S., V.S., R.K., and A.A. participated in material preparation. D.S., V.S., M.M., E.S., and T.B. contributed to computational analyses and pipelines. D.S., A.Y., and S.T.R. wrote the manuscript, with input from all other authors.

## Declaration of interests

S.T.R. is a co-founder and holds shares in Engimmune Therapeutics AG, Encelta, and Fy Cappa Biologics. S.T.R. holds shares in Alloy Therapeutics. S.T.R. serves on the scientific advisory board of Engimmune Therapeutics, Alloy Therapeutics, Encelta, and Fy Cappa Biologics. S.T.R. is a member of the board of directors for Engimmune Therapeutics. Authors V.S. and J.M.T. changed their institutional affiliations after completion of their contributions to the study. V.S. is now affiliated with the Institute of Science and Technology Austria (ISTA). J.M.T. is now affiliated with Engimmune Therapeutics.

## Declaration of generative AI and AI-assisted technologies in the writing process

During the preparation of this work, the authors used ChatGPT, Perplexity, and spell-check for language refinement and editing. After using these tools/services, the authors reviewed and edited the content as needed and take full responsibility for the content of the published article.

## STAR★Methods

### Key resources table

**Table:** 

REAGENT or RESOURCE	SOURCE	IDENTIFIER
**Antibodies**		

PE anti-DYKDDDDK Tag Antibody	Biolegend	Cat# 637309; RRID: AB_2563147
Alexa 647-conjugated Goat Anti-Human IgA+IgG + IgM	Jackson Immunoresearch	Cat# 109-605-064; RRID: AB_2337886

**Biological samples**		

COVID-19 vaccinated human serum samples	Anaea Trading SA	Cat# CUST-BB-11062021

**Critical commercial assays**		

Zymoprep Yeast Plasmid Miniprep II	Zymo Research	Cat# D2004
NEBridge® Golden Gate assembly kit	NEBridge®	Cat# E1601

**Deposited data**		

Raw sequencing data	This study	NCBI SRA: PRJNA1264489

**Experimental models: Organisms/strains**		

*S. cerevisiae* EBY100	ATCC	Cat# MYA-4941

**Oligonucleotides**		

NGS adaptor primers	Taft et al.^22^	Github: https://github.com/LSSI-ETH/Taft_Weber_2021

**Recombinant DNA**		

pYD1-RBD(wt)	Taft et al.^22^; derived from RRID:Addgene_73447	N/A
pYD1-GFP-dropout	This study	Zenodo: https://doi.org/10.5281/zenodo.18660254, Github: https://github.com/LSSI-ETH/Serum_DML_2025
RBD variant inserts	This study	Zenodo: https://doi.org/10.5281/zenodo.18660254, Github: https://github.com/LSSI-ETH/Serum_DML_2025

**Software and algorithms**		

bbduk	Github	Github: https://github.com/BioInfoTools/BBMap
R studio version 2023.03.0 + 386 (R version 4.3.1)	RStudio, Inc.	https://www.rstudio.com/
Data preprocessing, model training, and analysis code	This study	Zenodo: https://doi.org/10.5281/zenodo.18660254), Github: https://github.com/LSSI-ETH/Serum_DML_2025

### Experimental model and study participant details

#### Human serum samples

Serum samples collected under approval by the ethics committee of Sterling IRB (8291-BZhang) and purchased from RayBiotech through Anawa Trading SA (CUST-BB-11062021). Serum was obtained 6–18 days post vaccination with Pfizer-BioNTech or Moderna vaccine (Table S1). All serum samples were heat-inactivated at 56°C for 30 min.

### Method details

#### Serum pre-clearing

To remove yeast-binding serum antibodies, serum was mixed with washed EB100 cells in wash buffer (Dulbecco’s PBS+ 0.5% BSA +0.1% Tween 20 + 2 mM EDTA). The mixture was incubated for 1 h at 4°C with agitation (600–700 RPM). Yeast cells were pelleted (8000 x g, 30s) and the supernatant was collected and stored at 4°C until use. Pre-cleared serum was diluted with wash buffer to the desired concentration, such that the fluorescent signal from RBD binding was similar across all samples, while also minimizing background signal as assessed using the “No RBD” construct (yeast displaying all elements of the surface construct except the RBD).^12^

#### Screening RBD for serum binding and escape

ACE2 binding fractions of the previously described sublibraries 1C, 2C and 3C were pooled in equal ratios to form the 123C ACE2+ RBD library.^22^ Each sublibrary encodes the full SARS-CoV-2 RBD (201 amino acids) displayed on the yeast surface in a folded, functional state, preserving conformational epitopes for antibody binding. Only predefined stretches within the receptor-binding motif (RBM-1: residues 452–478, RBM-2: residues 484–505, RBM-3: residues 440–452) were mutagenized, while non-mutated positions within the RBD remained wild type. Thus, RBM mutations are assayed in the context of the folded full-length RBD.^13^^,^^22^ Cells were cultured in SD-UT medium (20 g/L glucose, 6.7 g/L yeast nitrogen base without amino acids, 5.4 g/L Na2HPO4, 8.6 g/L NaH2PO4·H2O and 5 g/L casamino acids) at 30°C in a 250 rpm shaking incubator. Surface expression was induced in SG-UT medium (20 g/L galactose, 6.7 g/L yeast nitrogen base without amino acids, 5.4 g/L Na2HPO4, 8.6 g/L NaH2PO4·H2O and 5 g/L casamino acids) at 23°C for 36–42 h in a 250 rpm shaking incubator.^41^ For each screen approximately 1.5 x 10^7^ library cells were washed twice with 1 mL of wash buffer (8000x g, 30 s) before staining. Primary staining was carried out with diluted serum samples, followed by secondary staining with anti-IgX (Alexa Fluor 647 AffiniPure Goat Anti-Human IgA + IgG + IgM (H + L), Jackson ImmunoResearch 109-605-064, 1:200 dilution), and tertiary staining with anti-Flag (PE-*anti*-DYKDDDDK Tag Antibody, Biolegend 637310, 1:200 dilution). Cells were washed twice in between stainings, and resuspended with wash buffer and kept on ice until FACS or flow cytometry. Cells were sorted by FACS (BD FACSAria Fusion) and flow cytometry analysis was performed on the BD Fortessa cytometer. Collected cells were pelleted and cultured in SD-UT media until desired OD.

#### Deep sequencing of RBD libraries

Deep sequencing was performed as previously described.^22^ In short: Plasmid DNA encoding the RBD variants was isolated following the manufacturer’s instructions (Zymo D2004). The region of interest was amplified using custom primers. Illumina Nextera barcode sequences were added in a second PCR amplification step, allowing for multiplexed high-throughput sequencing runs. Library preparation was performed using SPRIselect beads according to the manufacturer’s instructions (Beckman Coulter B23318). Populations were pooled at the desired ratios and sequenced using Illumina 2 x 250 PE protocols (NovaSeq instrument). For variant calling and all downstream analyses, the sequenced amplicon encompassed residues 417–505 (89 amino acids), which includes all RBM regions targeted by mutagenesis.

#### Experimental validation of selected RBD variants

RBD variant inserts were cloned into a pYD1 drop out plasmid, with a sfGFP in place of the RBD, using the NEBridge Golden Gate assembly kit according to manufacturer’s instructions (NEB #E1601). The assembled product was transformed into E. coli DH5α Mix & Go! Competent Cells competent cells (Zymo Research T3007) according to manufacturer’s instructions. sfGFP negative cell colonies were picked and cultured overnight. The pYD1 plasmids were purified using the Zymopure midiprep kit (Zymo Research, D4200). The plasmids were then transformed into EBY100 yeast using the Frozen-EZ Yeast Transformation kit (Zymo Research, T2001) according to the manufacturer’s instructions and plated on SD-UT agar. Single colonies were picked and cultured in SD-UT medium and subsequently screened using a BD Fortessa cytometer. Normalized serum reactivity was calculated by extracting the serum binding signal (anti-IgX) and RBD-expressing signal (anti-FLAG) for each cell of each variant and calculating the ratio between the two (anti-igX/anti-FLAG).

### Quantification and statistical analysis

#### Data processing

Data was processed as previously described.^22^ Reads were paired, quality trimmed and assembled using BBDuk on R. After extracting the region of interest (residues 417–505), sequences were translated using the R package Biostrings (v2.7.1). All sequences were filtered using a threshold of read counts >3. Moreover, sequences of incorrect length, sequences containing mutations outside of the library design and duplicate sequences were filtered out.

For machine learning analysis further filtering of sequences was carried out. For individual specific classifiers RBD sequences occurring in both the serum binding and serum-escape population within the set of sequences of the given individual were removed. For cohort-wide classifiers the individual labels were removed before the same filtering approach was applied.

#### Similarity calculations

Jaccard coefficients were defined as the size of the intersection divided by the size of the union of the RBD sequences recovered from each sorted fraction. Levenshtein distances were calculated compared to Wu-Hu-1 (YP_009724390.1).

#### RBM mutational analysis

Mutation frequencies across three receptor binding motifs (RBMs) within the SARS-CoV-2 RBD were calculated by comparing variant sequences to wildtype, weighted by sequence abundance, and stratified by serum binding status (binders vs. escape variants). Differential mutation frequency (Δf) was computed as the difference between escape and binder mutation frequencies at each position, with positive values indicating positions preferentially mutated in escape variants. To identify biologically significant positions, we applied a threshold of |Δf| ≥ 0.04, corresponding to the 75th percentile of the distribution and capturing the top quartile of differential mutational signatures. Additionally, we calculated a Chemical Switch Score for each position by classifying amino acids into four categories (positively charged, negatively charged, polar uncharged, and nonpolar) and quantifying whether mutations tended to preserve or change chemical properties, masked to positions exceeding the Δf threshold. Results were mapped onto the RBD structure (PDB: 6M0J, chain E) by replacing B-factors with Δf or chemical switch scores in the RBM positions, and visualized in PyMOL.

#### Machine learning model training and evaluation

##### Preprocessing

All code was built in Python (3.11.6) and data was prepared using Numpy (v1.23.5) and pandas (v1.4.2). Classifier performance was evaluated using accuracy, precision, recall, F1 score, and Matthews correlation coefficient (MCC). These metrics are defined as follows, with TP = true positives, TN = true negatives, FP = false positives, and FN = false negatives. All metrics were computed on held-out test data.

• Accuracy = (TP + TN)/(TP + TN + FP + FN)
• Precision = TP/(TP + FP)
• Recall = TP/(TP + FN)
• F1 score = 2 × (Precision × Recall)/(Precision + Recall)
• MCC = (TP × TN – FP × FN)/√((TP + FP)(TP + FN)(TN + FP)(TN + FN))

##### Logistic regression (LG), random forest (RF), recurrent neural network (RNN)

The data was split into a training and testing set using a 80/20 split. As not all sub libraries were equally represented in the dataset we balanced our training data by either upsampling the minority label class (binding/escape) within each RBM sublibrary, in the case of LG and RF using scikit-learn (v1.5). The long short-term memory (LSTM) recurrent neural networks (RNN) models were built using Keras and Tensorflow (v2.14.2), and data was balanced by weighing the majority class (binding/escape) within each RBM. Hyperparameter search was performed by doing a grid search (Table S4).

##### Transformer

Data was balanced using the same approach as for the RNN. Feature extraction was performed using ESM2-650M and embeddings were pooled in the sequence dimension using the “mean” pooling parameter from the PLMFit package.^25^ Embeddings were then fed into a logistic regression head. Hyperparameter tuning was performed according to the PLMFit package workflow.

#### Data visualization

Experimental workflows were done using Biorender. All other graphics were generated in R, using the packages ggplot2 (v3.4.2), ggpubr (v0.6.0) and ggseqlogo (v0.1), ggprism (v1.0.5). The packages readr (v2.1.4), reshape2 (v1.4.4), tidyverse (v2.0.0) were additionally used for data import and transformation. Experimental overviews were created using BioRender.com and final figures were assembled with Adobe illustrator.

## Supplemental information

Document S1. Figures S1–S6 and Tables S1–S4

Document S2. Article plus supplemental information

## References

[1] Tao K., Tzou P.L., Nouhin J., Gupta R.K., de Oliveira T., Kosakovsky Pond S.L., Fera D., Shafer R.W. (2021). The biological and clinical significance of emerging SARS-CoV-2 variants. Nat. Rev. Genet..

[2] Eguia R.T., Crawford K.H.D., Stevens-Ayers T., Kelnhofer-Millevolte L., Greninger A.L., Englund J.A., Boeckh M.J., Bloom J.D. (2021). A human coronavirus evolves antigenically to escape antibody immunity. PLoS Pathog..

[3] Hoffmann M., Kleine-Weber H., Schroeder S., Krüger N., Herrler T., Erichsen S., Schiergens T.S., Herrler G., Wu N.-H., Nitsche A. (2020). SARS-CoV-2 Cell Entry Depends on ACE2 and TMPRSS2 and Is Blocked by a Clinically Proven Protease Inhibitor. Cell.

[4] Piccoli L., Park Y.-J., Tortorici M.A., Czudnochowski N., Walls A.C., Beltramello M., Silacci-Fregni C., Pinto D., Rosen L.E., Bowen J.E. (2020). Mapping Neutralizing and Immunodominant Sites on the SARS-CoV-2 Spike Receptor-Binding Domain by Structure-Guided High-Resolution Serology. Cell.

[5] Greaney A.J., Loes A.N., Gentles L.E., Crawford K.H.D., Starr T.N., Malone K.D., Chu H.Y., Bloom J.D. (2021). Antibodies elicited by mRNA-1273 vaccination bind more broadly to the receptor binding domain than do those from SARS-CoV-2 infection. Sci. Transl. Med..

[6] Barnes C.O., West A.P., Huey-Tubman K.E., Hoffmann M.A.G., Sharaf N.G., Hoffman P.R., Koranda N., Gristick H.B., Gaebler C., Muecksch F. (2020). Structures of human antibodies bound to SARS-CoV-2 spike reveal common epitopes and recurrent features of antibodies. Cell.

[7] Barnes C.O., Jette C.A., Abernathy M.E., Dam K.-M.A., Esswein S.R., Gristick H.B., Malyutin A.G., Sharaf N.G., Huey-Tubman K.E., Lee Y.E. (2020). SARS-CoV-2 neutralizing antibody structures inform therapeutic strategies. Nature.

[8] Cameroni E., Bowen J.E., Rosen L.E., Saliba C., Zepeda S.K., Culap K., Pinto D., VanBlargan L.A., De Marco A., di Iulio J. (2022). Broadly neutralizing antibodies overcome SARS-CoV-2 Omicron antigenic shift. Nature.

[9] Ma W., Fu H., Jian F., Cao Y., Li M. (2023). Immune evasion and ACE2 binding affinity contribute to SARS-CoV-2 evolution. Nat. Ecol. Evol..

[10] Cao Y., Wang J., Jian F., Xiao T., Song W., Yisimayi A., Huang W., Li Q., Wang P., An R. (2022). Omicron escapes the majority of existing SARS-CoV-2 neutralizing antibodies. Nature.

[11] Lopez-Morales J., Vanella R., Utzinger T., Schittny V., Hirsiger J., Osthoff M., Berger C.T., Guri Y., Nash M.A. (2023). Multiplexed on-yeast serological assay for immune escape screening of SARS-CoV-2 variants. iScience.

[12] Greaney A.J., Loes A.N., Crawford K.H.D., Starr T.N., Malone K.D., Chu H.Y., Bloom J.D. (2021). Comprehensive mapping of mutations in the SARS-CoV-2 receptor-binding domain that affect recognition by polyclonal human plasma antibodies. Cell Host Microbe.

[13] Starr T.N., Greaney A.J., Hilton S.K., Ellis D., Crawford K.H.D., Dingens A.S., Navarro M.J., Bowen J.E., Tortorici M.A., Walls A.C. (2020). Deep Mutational Scanning of SARS-CoV-2 Receptor Binding Domain Reveals Constraints on Folding and ACE2 Binding. Cell.

[14] Starr T.N., Greaney A.J., Dingens A.S., Bloom J.D. (2021). Complete map of SARS-CoV-2 RBD mutations that escape the monoclonal antibody LY-CoV555 and its cocktail with LY-CoV016. Cell Rep. Med..

[15] Starr T.N., Greaney A.J., Addetia A., Hannon W.W., Choudhary M.C., Dingens A.S., Li J.Z., Bloom J.D. (2021). Prospective mapping of viral mutations that escape antibodies used to treat COVID-19. Science.

[16] Starr T.N., Czudnochowski N., Liu Z., Zatta F., Park Y.-J., Addetia A., Pinto D., Beltramello M., Hernandez P., Greaney A.J. (2021). SARS-CoV-2 RBD antibodies that maximize breadth and resistance to escape. Nature.

[17] Greaney A.J., Starr T.N., Barnes C.O., Weisblum Y., Schmidt F., Caskey M., Gaebler C., Cho A., Agudelo M., Finkin S. (2021). Mapping mutations to the SARS-CoV-2 RBD that escape binding by different classes of antibodies. Nat. Commun..

[18] Greaney A.J., Starr T.N., Gilchuk P., Zost S.J., Binshtein E., Loes A.N., Hilton S.K., Huddleston J., Eguia R., Crawford K.H.D. (2021). Complete mapping of mutations to the SARS-CoV-2 spike receptor-binding domain that escape antibody recognition. Cell Host Microbe.

[19] Starr T.N., Greaney A.J., Hannon W.W., Loes A.N., Hauser K., Dillen J.R., Ferri E., Farrell A.G., Dadonaite B., McCallum M. (2022). Shifting mutational constraints in the SARS-CoV-2 receptor-binding domain during viral evolution. Science.

[20] Cao Y., Jian F., Wang J., Yu Y., Song W., Yisimayi A., Wang J., An R., Chen X., Zhang N. (2023). Imprinted SARS-CoV-2 humoral immunity induces convergent Omicron RBD evolution. Nature.

[21] Fowler D.M., Fields S. (2014). Deep mutational scanning: a new style of protein science. Nat. Methods.

[22] Taft J.M., Weber C.R., Gao B., Ehling R.A., Han J., Frei L., Metcalfe S.W., Overath M.D., Yermanos A., Kelton W., Reddy S.T. (2022). Deep mutational learning predicts ACE2 binding and antibody escape to combinatorial mutations in the SARS-CoV-2 receptor-binding domain. Cell.

[23] Ehling R.A., Minot M., Overath M.D., Sheward D.J., Han J., Gao B., Taft J.M., Pertseva M., Weber C.R., Frei L. (2025). Synthetic coevolution reveals adaptive mutational trajectories of neutralizing antibodies and SARS-CoV-2. Cell Syst.

[24] Frei L., Gao B., Han J., Taft J.M., Irvine E.B., Weber C.R., Kumar R.K., Eisinger B.N., Ignatov A., Yang Z., Reddy S.T. (2025). Deep mutational learning for the selection of therapeutic antibodies resistant to the evolution of Omicron variants of SARS-CoV-2. Nat. Biomed. Eng..

[25] Bikias T., Stamkopoulos E., Reddy S.T. (2025). PLMFit: benchmarking transfer learning with protein language models for protein engineering. Brief. Bioinform..

[26] Traxlmayr M.W. (2022).

[27] Glick M., Klon A.E., Acklin P., Davies J.W. (2003). Prioritization of high throughput screening data of compound mixtures using molecular similarity. Mol. Phys..

[28] Minot M., Reddy S.T. (2024). Meta learning addresses noisy and under-labeled data in machine learning-guided antibody engineering. Cell Syst..

[29] Lee J.M., Eguia R., Zost S.J., Choudhary S., Wilson P.C., Bedford T., Stevens-Ayers T., Boeckh M., Hurt A.C., Lakdawala S.S. (2019). Mapping person-to-person variation in viral mutations that escape polyclonal serum targeting influenza hemagglutinin. eLife.

[30] Dejnirattisai W., Zhou D., Ginn H.M., Duyvesteyn H.M.E., Supasa P., Case J.B., Zhao Y., Walter T.S., Mentzer A.J., Liu C. (2021). The antigenic anatomy of SARS-CoV-2 receptor binding domain. Cell.

[31] Yuan M., Huang D., Lee C.-C.D., Wu N.C., Jackson A.M., Zhu X., Liu H., Peng L., van Gils M.J., Sanders R.W. (2021). Structural and functional ramifications of antigenic drift in recent SARS-CoV-2 variants. Science.

[32] Amanat F., Thapa M., Lei T., Ahmed S.M.S., Adelsberg D.C., Carreño J.M., Strohmeier S., Schmitz A.J., Zafar S., Zhou J.Q. (2021). SARS-CoV-2 mRNA vaccination induces functionally diverse antibodies to NTD, RBD, and S2. Cell.

[33] Lima N.S., Musayev M., Johnston T.S., Wagner D.A., Henry A.R., Wang L., Yang E.S., Zhang Y., Birungi K., Black W.P. (2022). Primary exposure to SARS-CoV-2 variants elicits convergent epitope specificities, immunoglobulin V gene usage and public B cell clones. Nat. Commun..

[34] Chen E.C., Gilchuk P., Zost S.J., Suryadevara N., Winkler E.S., Cabel C.R., Binshtein E., Chen R.E., Sutton R.E., Rodriguez J. (2021). Convergent antibody responses to the SARS-CoV-2 spike protein in convalescent and vaccinated individuals. Cell Rep..

[35] Yan Q., Gao X., Liu B., Hou R., He P., Ma Y., Zhang Y., Zhang Y., Li Z., Chen Q. (2024). Antibodies utilizing VL6-57 light chains target a convergent cryptic epitope on SARS-CoV-2 spike protein and potentially drive the genesis of Omicron variants. Nat. Commun..

[36] Dadonaite B., Burrell A.R., Logue J., Chu H.Y., Payne D.C., Haslam D.B., Staat M.A., Bloom J.D. (2025). SARS-CoV-2 neutralizing antibody specificities differ dramatically between recently infected infants and immune-imprinted individuals. J. Virol..

[37] Abebe E.C., Dejenie T.A. (2023). Protective roles and protective mechanisms of neutralizing antibodies against SARS-CoV-2 infection and their potential clinical implications. Front. Immunol..

[38] Zhang A., Stacey H.D., D’Agostino M.R., Tugg Y., Marzok A., Miller M.S. (2023). Beyond neutralization: Fc-dependent antibody effector functions in SARS-CoV-2 infection. Nat. Rev. Immunol..

[39] Greaney A.J., Starr T.N., Bloom J.D. (2021). An antibody-escape calculator for mutations to the SARS-CoV-2 receptor-binding domain. bioRxiv.

[40] Markov P.V., Ghafari M., Beer M., Lythgoe K., Simmonds P., Stilianakis N.I., Katzourakis A. (2023). The evolution of SARS-CoV-2. Nat. Rev. Microbiol..

[41] Boder E.T., Wittrup K.D. (1997). Yeast surface display for screening combinatorial polypeptide libraries. Nat. Biotechnol..

