# Supplementary material for: Dissecting serum polyclonal antibody escape to SARS-CoV-2 variants by deep mutational learning
Source: Cell Rep Methods. 2026 Apr 23;6(6):101417. doi: 10.1016/j.crmeth.2026.101417 (PMC13282657; doi:10.1016/j.crmeth.2026.101417)
Supplement: Document S1. Figures S1–S6 and Tables S1–S4 [file mmc1.pdf]

Cell Reports Methods, Volume *6*

Supplemental information

Dissecting serum polyclonal antibody escape to SARS-CoV-2 variants by deep mutational learning

Danielle Shlesinger, Viktor Sadilek, Mason Minot, Evangelos Stamkopoulos, Thomas Bikias, Raphael Kuhn, Andreas Agrafiotis, Joseph M. Taft, Alexander Yermanos, and Sai.T. Reddy

Supplementary Data

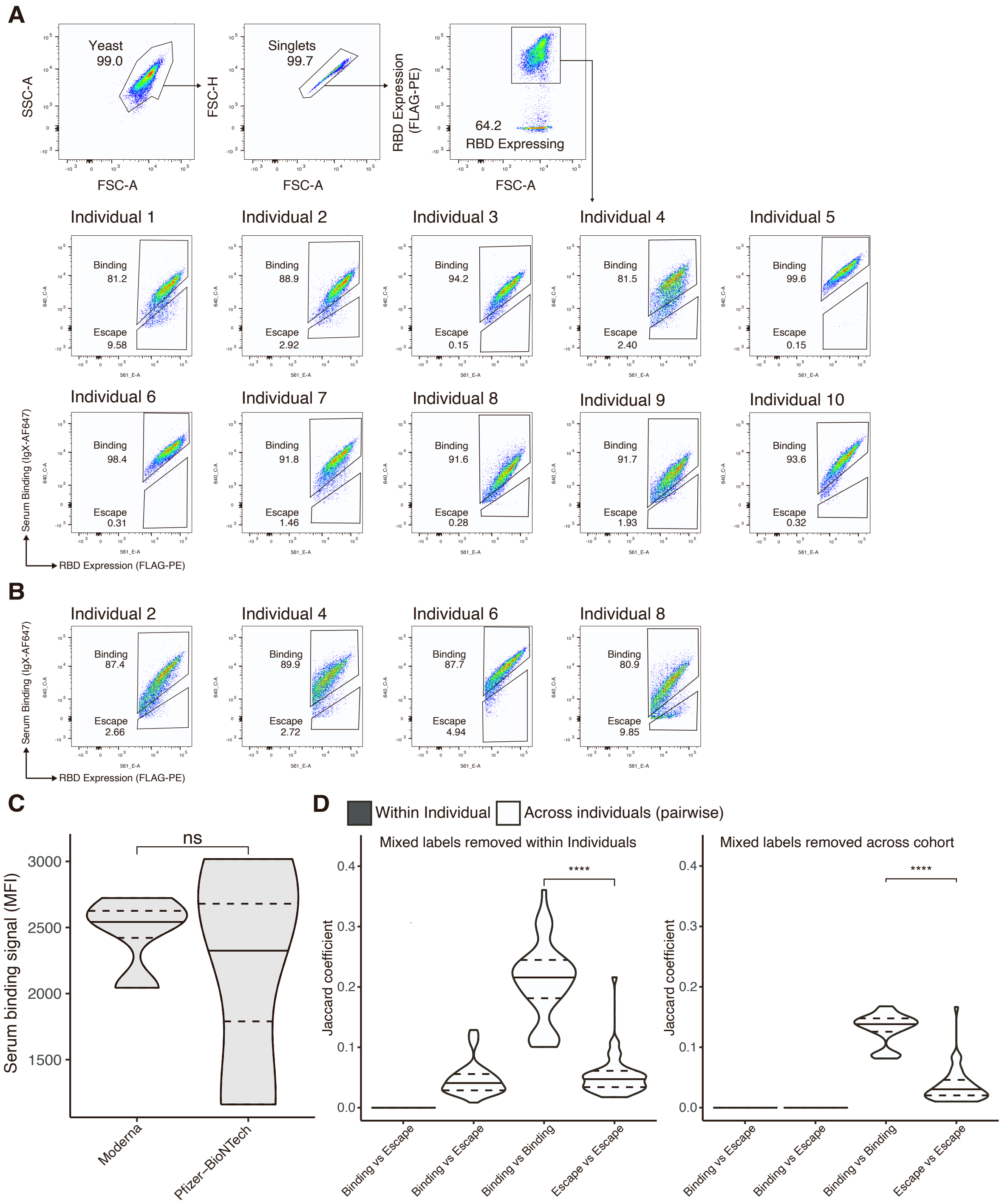

Figure S1. Yeast display screening of combinatorial RBD libraries for serum binding and escape. Related to Figure 1. A) Control RBD (Wu-Hu-1) gating schemes used for selection of serum binding and -escape variants. B) Flow cytometry dot plots depicting screening of ACE2+ 123C library against serum of the individuals not included in Figure 1. Approximately 1.5 x 10^7 yeast cells were screened for each individual serum. C) Violin plot depicting difference in serum binding signal (MFI) between vaccine types. D) Violin plot depicting Jaccard index between binding and escape fractions within and across individuals. Sequences with mixed labels were removed on an individual level (left) or cohort level (right). $*p<0.05$, $**p<0.01$, $***p<0.001$, $****p<0.0001$, not significant (ns).

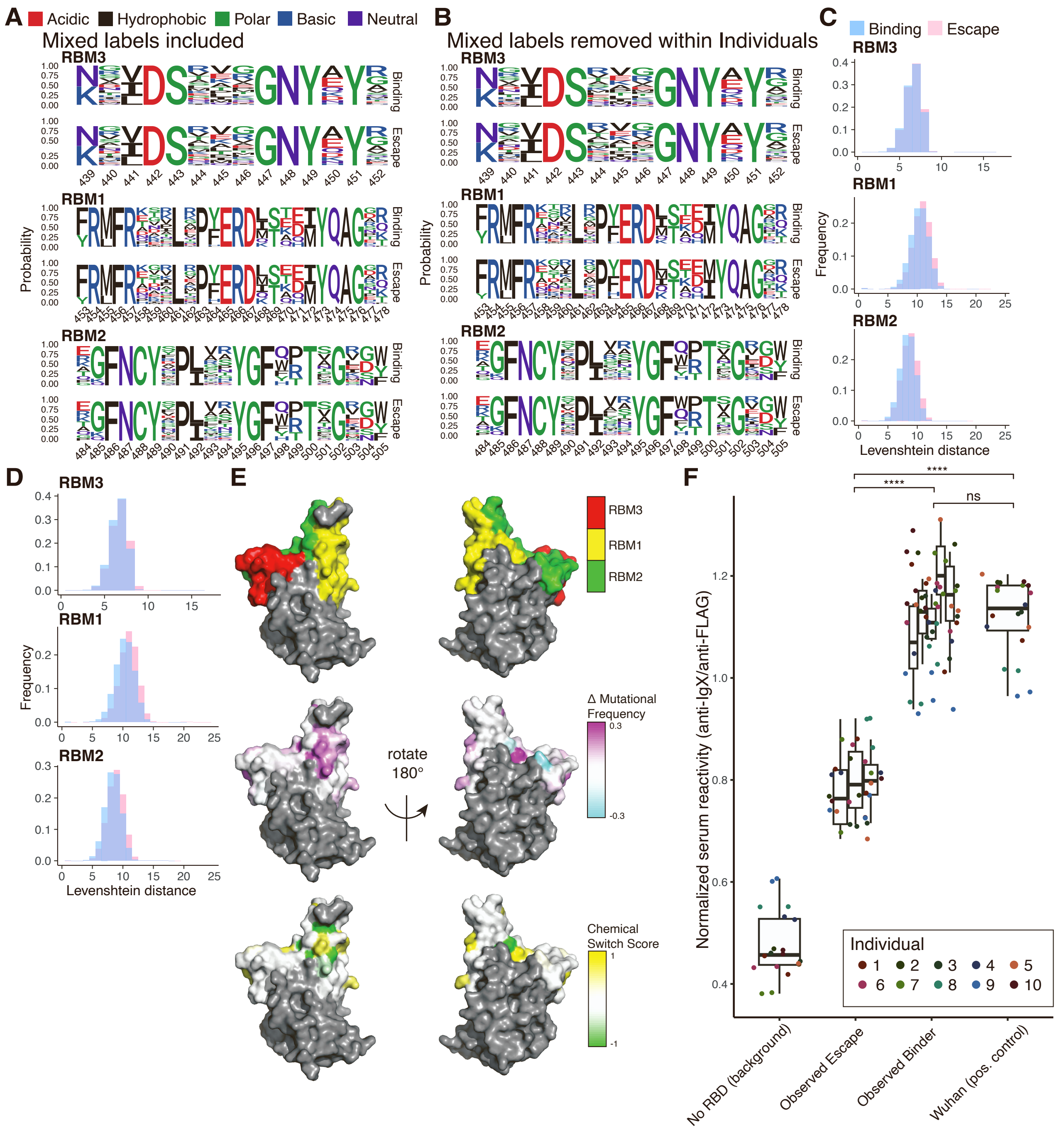

Figure S2. Additional characterization overlap, diversity and sequence similarity between serum binders and escape. Related to Figure 2 A) and B) RBM sequence logo plots from serum-binding and -escape variants (left) and their comparison of distributions of mutational distances of RBMs and comparison of RBM diversity at each mutational distance (right). Sequences with mixed labels were included (C) or removed on an individual level (D). Mutational distance was measured as Levenshtein distance of an RBM from the Wu-Hu-1 sequence. E) Structure of the SARS-CoV-2 RBD (PDB: 6M0J, chain E) colored by RBM regions (top), differential mutation frequency (Δf = escape frequency − binder frequency) (middle), and chemical switch score at positions with significant differential mutation ($|\Delta f| \geq 0.04$). The chemical switch score quantifies whether mutations tend to alter charge, polarity, or hydrophobicity versus making conservative substitutions within the same chemical category. F) Group comparison of normalized serum reactivity (mean signal ratio anti-IgX/anti-Flag ratios (anti-IgX/anti-Flag) for observed serum binding and -escape variants for all 8 tested observed variants. Statistical analysis was performed across groups using the unpaired two-tailed Student's t test and is shown for observed binders vs escape and relative to Wu-Hu-1. "No RBD" serves as a background reference. By design, all groups are significantly above "No RBD"; full pairwise statistics including "No RBD" comparisons are provided in Table S3. $*p<0.05$, $**p<0.01$, $***p<0.001$, $****p<0.0001$, not significant (ns).

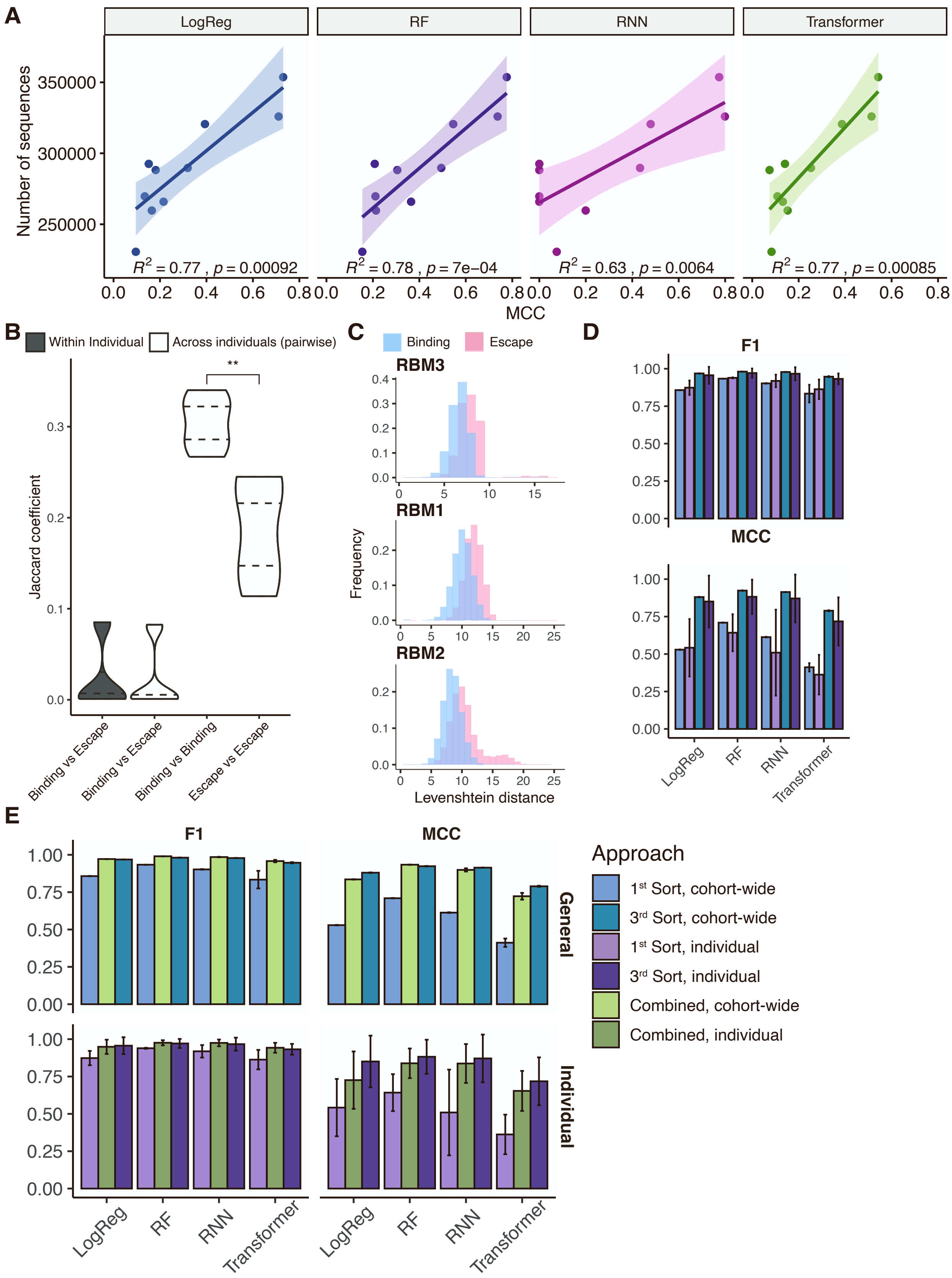

Figure S3. Correlation between training data, screening and machine learning model performance. Related to Figure 3. A) Dot plots showing the correlation between MCC scores of individual classifiers and number of sequences retrieved for the corresponding individual. B) Violin plot depicting Jaccard coefficient between enriched binding and enriched escape fractions within and across individuals. C) Comparison of distributions of mutational distances of RBMs from serum binding and escape fractions and comparison of RBM diversity at each mutational distance of serum binding

and escape variants. Mutational distance was measured as Levenshtein distance of an RBM from the Wu-Hu-1 sequence. D) Comparison of performance metrics across averages of different individual-specific classifiers or a cohort classifier (logistic model [LM], random forest [RF], recurrent neural network [RNN]), Transformer) trained on three times sorted and once-sorted data. E) Comparison of performance metrics across averages of different individual specific classifiers or a cohort classifier (logistic model [LM], random forest [RF], recurrent neural network [RNN]), Transformer) trained on once-sorted binding and three times sorted escape fractions. Standard deviations are shown by error bars and represent training across individuals and random-seed runs. Statistical analysis was performed across groups using the unpaired two-tailed Student's t test. $*p<0.05$, $**p<0.01$, $***p<0.001$, $****p<0.0001$, not significant (ns).

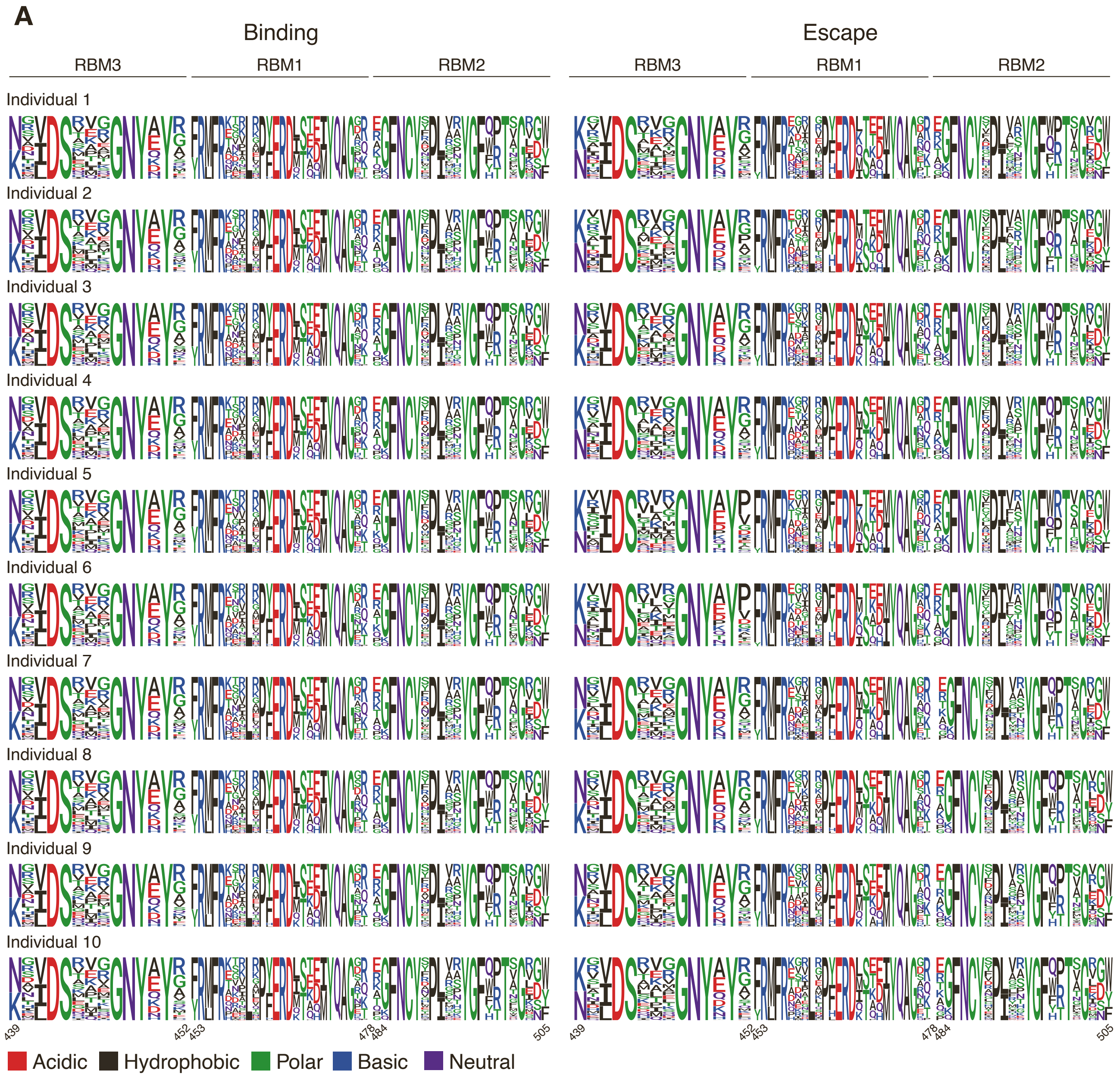

Figure S4. Individual RBM protein sequence logo plots from serum-binding and -escape variants. Related to Figure 4. A) RBM sequence logo plots from serum-binding and -escape variants. Sequences with mixed labels were removed on an individual level.

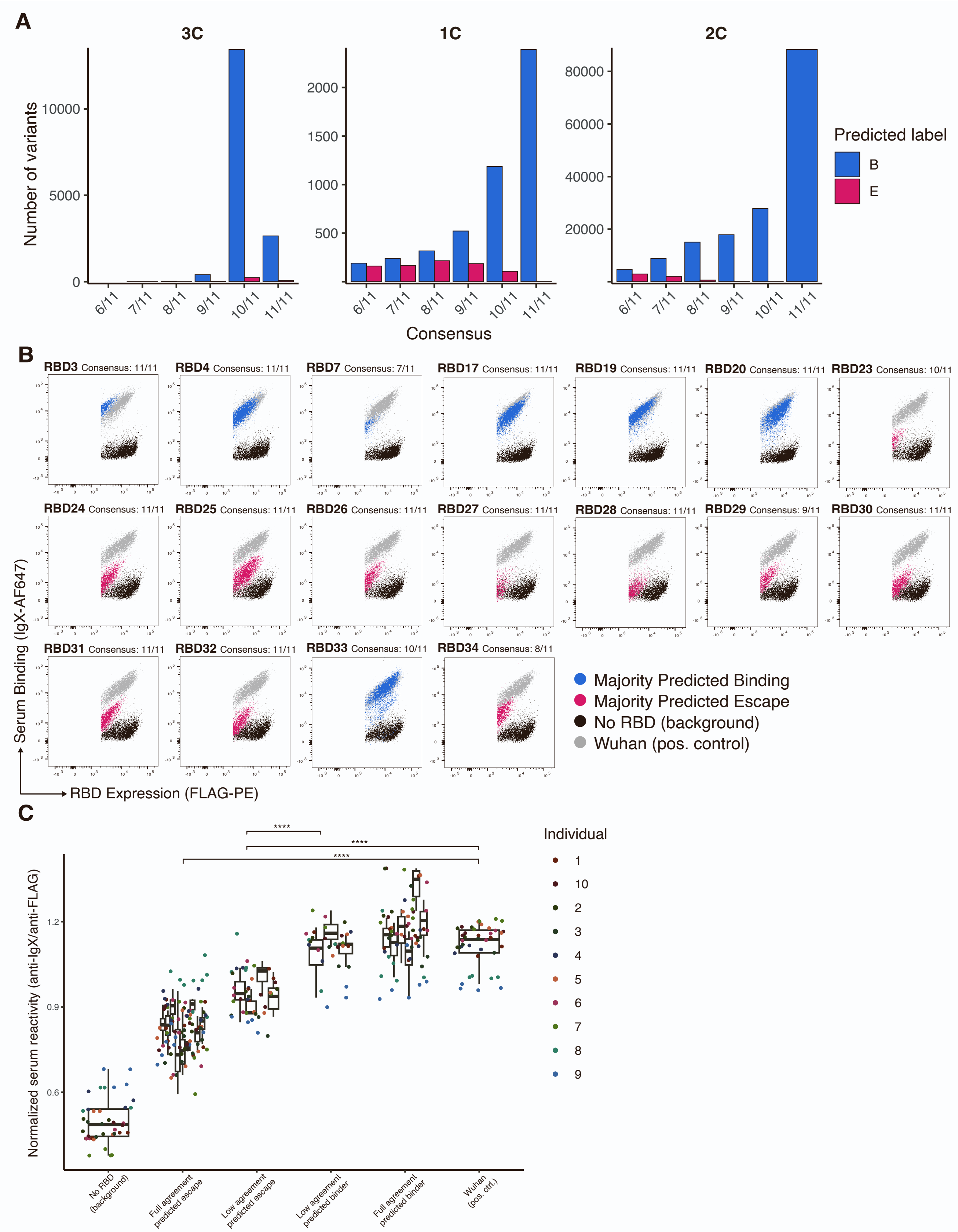

Figure S5. Consensus of trained models is consistent with experimental validations. Related to Figure 4. A) Bar plots showing the degree of agreement between trained classifiers on unseen RBD variants (not present in training or test data). B) Rest of representative FACS plots from screening serum against unseen RBD variants, where the ensemble of all models had full agreement. Gating strategy as described in Figure 1B. C) Comparison of normalized serum reactivities (anti-IgX/anti-Flag) for predicted serum binding- and -escape variants with either full or low ensemble model agreement.

Statistical analysis was performed using the unpaired two-tailed Student's t test and is shown for predicted binders vs escape and relative to the Wu-Hu-1 positive control. "No RBD" serves as a background reference. By design, all groups are significantly above "No RBD"; full pairwise statistics including "No RBD" comparisons are provided in Table 3. $*p<0.05$, $**p<0.01$, $***p<0.001$, $****p<0.0001$, not significant (ns).

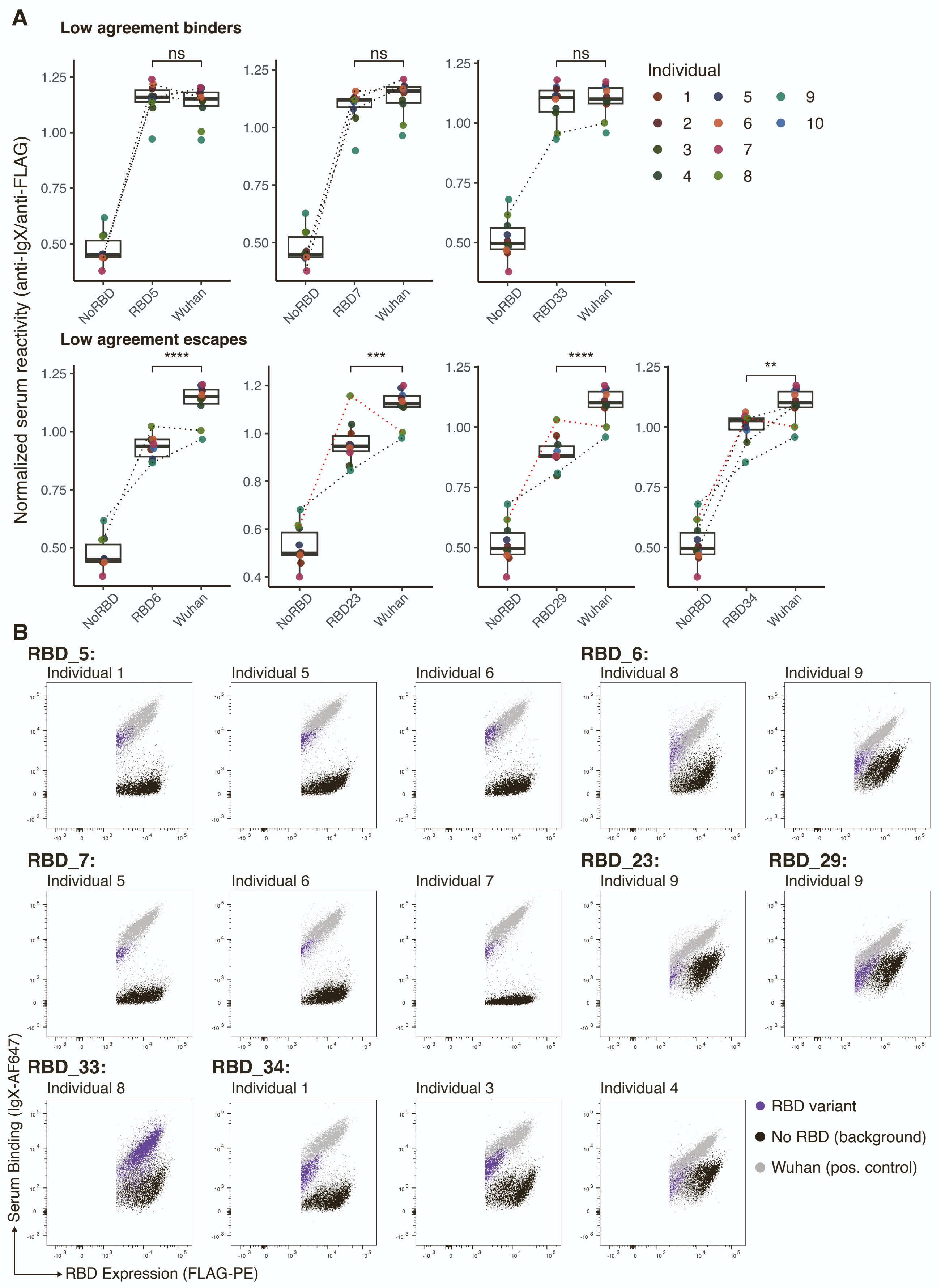
A
Low agreement binders
Low agreement escapes
Normalized serum reactivity (anti-IgX/anti-FLAG)
ns
****
***
**
NoRBD
RBD5
RBD7
RBD33
RBD6
RBD23
RBD29
RBD34
Wuhan
Individual
B
RBD_5:
RBD_6:
RBD_7:
RBD_23:
RBD_29:
RBD_33:
RBD_34:
Individual 1
Individual 3
Individual 4
Individual 5
Individual 6
Individual 7
Individual 8
Individual 9
Serum Binding (IgX-AF647)
RBD Expression (FLAG-PE)
RBD variant
No RBD (background)
Wuhan (pos. control)

Figure S6. Majority of low agreement predictions are consistent with experimental validations. Related to Figure 4. A) Comparison of normalized serum reactivity (anti-IgX/anti-Flag) for predicted serum binding- and escape-variants with low ensemble model agreement. Black dotted lines represent minority predictions and red dotted lines represent majority predictions that defy the trend. B) FACS plots from screening serum against RBD variants. The shown individuals and RBD variants represent the disagreement of the minority of the consensus predictions. Gating strategy as described in Figure 1B. Dotted lines represent minority predictions or majority predictions that are incorrect for the specific individual. Statistical analysis was performed using the unpaired two-tailed Student's t test. "No RBD" serves as a background reference. By design, RBDs are above "No RBD";. $*p<0.05$, $**p<0.01$, $***p<0.001$, $****p<0.0001$, not significant (ns).

Individual	Sample ID	Sex	Days post 2nd vaccine	Vaccine Manufacturer	Serum Dilution
1	V974C8	Female	8	Pfizer-BioNTech	1 : 400
2	V985C7	Male	7	Moderna	1 : 400
3	V977C10	Female	10	Pfizer-BioNTech	1 : 800
4	V981C	Male	15	Moderna	1 : 400
5	V979C8	Female	8	Pfizer-BioNTech	1 : 200
6	V967C	Male	17	Moderna	1 : 100
7	V980C9	Female	9	Pfizer-BioNTech	1 : 400
8	V989C	Male	6	Pfizer-BioNTech	1 : 400
9	V964C9	Female	9	Pfizer-BioNTech	1 : 400
10	V952C	Male	18	Moderna	1 : 400

Table S1. Metadata for individual serum samples used in this study. Related to STAR Methods. Individual is the simplified identifier used in this study, sample ID is the unique identifier given by vendor (RayBiotech), serum dilution refers to the specific dilution level at which each experiment was conducted, days post second vaccination indicated the sample collection time point, sex and type of mRNA vaccine is also indicated.

Variant	Observed label	Predicted majority label	Sub-library	LV	Sequence	Consensus	Individual Model Prediction (Low Agreement)
RBD_9	Binding	NA	1C	11	KIADYNYKLPD DFTGCVIAWNS NNLDSKVGGN YNYLFRMFRK ARLRPYERDKT EEIYQAGAKPC NGVEGFNCYFP LQSYGFQPTNG VGY	NA	NA
RBD_10	Binding	NA	3C	6	KIADYNYKLPD DFTGCVIAWNS NEVDSQEGGN YDYVYRLFRKS NLKPFERDISTE IYQAGSTPCNG VEGFNCYFPLQ SYGFQPTNGVG Y	NA	NA
RBD_11	Binding	NA	3C	6	KIADYNYKLPD DFTGCVIAWNS NTIDSRPGGNY	NA	NA

					EYGYRLFRKSN<br>LKPFERDISTEI<br>YQAGSTPCNGV<br>EGFNCYFPLQS<br>YGFQPTNGVGY		
RBD_12	Binding	NA	3C	4	KIADYNYKLPD<br>DFTGCVIAWNS<br>NGVDSEVGGN<br>YKYLYRLFRKS<br>NLKPFERDISTE<br>IYQAGSTPCNG<br>VEGFNCYFPLQ<br>SYGFQPTNGVG<br>Y	NA	NA
RBD_13	Escape	NA	1C	12	KIADYNYKLPD<br>DFTGCVIAWNS<br>NNLDSKVGGN<br>YNYLFRMFRPR<br>ILVPLERDKTTE<br>MYQAGNKPCN<br>GVEGFNCYFPL<br>QSYGFQPTNGV<br>GY	NA	NA
RBD_14	Escape	NA	3C	7	KIADYNYKLPD<br>DFTGCVIAWNS<br>NTVDSVEMGN<br>YKYPYRLFRKS<br>NLKPFERDISTE<br>IYQAGSTPCNG<br>VEGFNCYFPLQ<br>SYGFQPTNGVG<br>Y	NA	NA
RBD_15	Escape	NA	3C	6	KIADYNYKLPD<br>DFTGCVIAWNS<br>NLIDSKIMGNY<br>TYPYRLFRKSN<br>LKPFERDISTEI<br>YQAGSTPCNGV<br>EGFNCYFPLQS<br>YGFQPTNGVGY	NA	NA
RBD_21	Binding	NA	2C	8	KIADYNYKLPD<br>DFTGCVIAWNS<br>NNLDSKVGGN<br>YNYLYRLFRKS<br>NLKPFERDISTE<br>IYQAGSTPCNG<br>VEGFNCYRPIA<br>SYGFQRTYGQD<br>F	NA	NA
RBD_1	NA	Binding	3C	1	KIADYNYKLPD<br>DFTGCVIAWNS<br>NQLDSKVGGN<br>YNYLYRLFRKS<br>NLKPFERDISTE<br>IYQAGSTPCNG<br>VEGFNCYFPLQ<br>SYGFQPTNGVG<br>Y	11/11	NA

RBD_3	NA	Binding	2C	10	KIADYNYKLPDDFTGCVIAWNSNNLDSKVGGNYNYLYRLFRKSNLKPFERDISTEIYQAGSTPCNGVQGFNCYSPIRRYGFWPTIGDSW	11/11	NA	
RBD_4	NA	Binding	1C	5	KIADYNYKLPDDFTGCVIAWNSNNLDSKVGGNYNYLYRMFRESILVPFERDISTEIYQAGGTPCNGVEGFNCYFPLQSYGFQPTNGVGY	11/11	NA	
RBD_5	NA	Binding	1C	10	KIADYNYKLPDDFTGCVIAWNSNNLDSKVGGNYNYLFRMFRKRILIPYERDQTTHMYQAGSTPCNGVEGFNCYFPLQSYGFQPTNGVGY	8/11	Cohort-wide<br>Individual 1<br>Individual 2<br>Individual 3<br>Individual 4<br>Individual 5<br>Individual 6<br>Individual 7<br>Individual 8<br>Individual 9<br>Individual 10	Binding<br>Escape<br>Binding<br>Binding<br>Binding<br>Escape<br>Escape<br>Binding<br>Binding<br>Binding<br>Binding
RBD_6	NA	Escape	1C	14	KIADYNYKLPDDFTGCVIAWNSNNLDSKVGGNYNYLFRMFRAVALAPYERDKTADMYQAGAKPCNGVEGFNCYFPLQSYGFQPTNGVGY	9/11	Cohort-wide<br>Individual 1<br>Individual 2<br>Individual 3<br>Individual 4<br>Individual 5<br>Individual 6<br>Individual 7<br>Individual 8<br>Individual 9<br>Individual 10	Escape<br>Escape<br>Escape<br>Escape<br>Escape<br>Escape<br>Escape<br>Escape<br>Binding<br>Binding<br>Escape
RBD_7	NA	Binding	2C	6	KIADYNYKLPDDFTGCVIAWNSNNLDSKVGGNYNYLYRLFRKSNLKPFERDISTEIYQAGSTPCNGVEKFNCYFPIFS	7/11	Cohort-wide<br>Individual 1<br>Individual 2<br>Individual 3<br>Individual 4	Escape<br>Binding<br>Binding<br>Binding<br>Binding

					YGFQRTAGMGY		Individual 5<br>Individual 6<br>Individual 7<br>Individual 8<br>Individual 9<br>Individual 10	Escape<br>Escape<br>Escape<br>Binding<br>Binding<br>Binding
RBD_8	NA	Escape	3C	5	KIADYNYKLPDDFTGCVIAWNSNTLDSKLQGNYTYPYRLFRKSNLKPFERDISTEIYQAGSTPCNGVEGFNCYFPLQSYGFQPTNGVGY	11/11	NA	
RBD_17	NA	Binding	1C	9	KIADYNYKLPDDFTGCVIAWNSNNLDSKVGGNYNYLFRMFRHGNLAPYERDITTQIYQAGETPCNGVEGFNCYFPLQSYGFQPTNGVGY	11/11	NA	
RBD_19	NA	Binding	1C	8	KIADYNYKLPDDFTGCVIAWNSNNLDSKVGGNYNYLFRMFRTGTLRPYERDISAEIYQAGTKPCNGVEGFNCYFPLQSYGFQPTNGVGY	11/11	NA	
RBD_20	NA	Binding	1C	10	KIADYNYKLPDDFTGCVIAWNSNNLDSKVGGNYNYLYRMFRKSALMPFERDMTAEIYQAGDTPCNGVEGFNCYFPLQSYGFQPTNGVGY	11/11	NA	
RBD_23	NA	Escape	2C	10	KIADYNYKLPDDFTGCVIAWNSNNLDSKVGGNYNYLYRLFRKSNLKPFERDISTEIYQAGSTPCNGVEQFNCYCPIHSYGFHRTTGQDW	10/11	Cohort-wide<br>Individual 1<br>Individual 2<br>Individual 3<br>Individual 4<br>Individual 5<br>Individual 6<br>Individual 7<br>Individual 8<br>Individual 9	Escape<br>Escape<br>Escape<br>Escape<br>Escape<br>Escape<br>Escape<br>Escape<br>Escape<br>Binding

							Individual 10	Escape
RBD_24	NA	Escape	3C	8	KIADYNYKLPDDFTGCVIAWNSKEIDSGAAGNYAYPYRLFRKSNLKPFERDISTEIYQAGSTPCNGVEGFNCYFPLQSYGFQPTNGVGY	11/11	NA	
RBD_25	NA	Escape	1C	12	KIADYNYKLPDDFTGCVIAWNSNNLDSKVGGNYNYLFRMFRNESLGPHERDKTTHIYQAGTKPCNGVEGFNCYFPLQSYGFQPTNGVGY	11/11	NA	
RBD_26	NA	Escape	3C	5	KIADYNYKLPDDFTGCVIAWNSNNLDSNLPGNYAYPYRLFRKSNLKPFERDISTEIYQAGSTPCNGVEGFNCYFPLQSYGFQPTNGVGY	11/11	NA	
RBD_27	NA	Escape	3C	6	KIADYNYKLPDDFTGCVIAWNSKAIDSDVTGNYNYPYRLFRKSNLKPFERDISTEIYQAGSTPCNGVEGFNCYFPLQSYGFQPTNGVGY	11/11	NA	
RBD_28	NA	Escape	3C	7	KIADYNYKLPDDFTGCVIAWNSKAIDSQVMGNYEYPYRLFRKSNLKPFERDISTEIYQAGSTPCNGVEGFNCYFPLQSYGFQPTNGVGY	11/11	NA	
RBD_29	NA	Escape	2C	12	KIADYNYKLPDDFTGCVIAWNSNNLDSKVGGNYNYLYRLFRKSNLKPFERDISTEIYQAGSTPCNGVRKFNCYRPIGEYGFHTTTGESW	10/11	Cohort-wide	Escape
							Individual 1	Escape
							Individual 2	Escape
							Individual 3	Escape
							Individual 4	Escape
							Individual 5	Escape
							Individual 6	Escape
							Individual 7	Escape
							Individual 8	Escape
							Individual 9	Binding

							Individual 10	Escape
RBD_30	NA	Escape	3C	8	KIADYNYKLPD<br>DFTGCVIAWNS<br>KEIDSMTRGNY<br>AYPYRLFRKSN<br>LKPFERDISTEI<br>YQAGSTPCNGV<br>EGFNCYFPLQS<br>YGFQPTNGVGY	11/11	NA	
RBD_31	NA	Escape	3C	7	KIADYNYKLPD<br>DFTGCVIAWNS<br>NEVDSPLHGNY<br>PYPYRLFRKSN<br>LKPFERDISTEI<br>YQAGSTPCNGV<br>EGFNCYFPLQS<br>YGFQPTNGVGY	11/11	NA	
RBD_32	NA	Escape	3C	6	KIADYNYKLPD<br>DFTGCVIAWNS<br>NTVDSMVNGN<br>YKYPYRLFRKS<br>NLKPFERDISTE<br>IYQAGSTPCNG<br>VEGFNCYFPLQ<br>SYGFQPTNGVG<br>Y	11/11	NA	
RBD_33	NA	Binding	3C	7	KIADYNYKLPD<br>DFTGCVIAWNS<br>KAIDSAEGGNY<br>DYRYRLFRKSN<br>LKPFERDISTEI<br>YQAGSTPCNGV<br>EGFNCYFPLQS<br>YGFQPTNGVGY	10/11	Cohort-wide<br>Individual 1<br>Individual 2<br>Individual 3<br>Individual 4<br>Individual 5<br>Individual 6<br>Individual 7<br>Individual 8<br>Individual 9<br>Individual 10	Binding<br>Binding<br>Binding<br>Binding<br>Binding<br>Binding<br>Binding<br>Binding<br>Escape<br>Binding<br>Binding
RBD_34	NA	Escape	1C	9	KIADYNYKLPD<br>DFTGCVIAWNS<br>NNLDSKVGGN<br>YNYLFRLFRDV<br>DLMPFERDITE<br>DMYQAGSKPC<br>NGVEGFNCYFP<br>LQSYGFQPTNG<br>VGY	8/11	Cohort-wide<br>Individual 1<br>Individual 2<br>Individual 3<br>Individual 4<br>Individual 5<br>Individual 6<br>Individual 7<br>Individual 8<br>Individual 9<br>Individual 10	Escape<br>Escape<br>Escape<br>Binding<br>Binding<br>Escape<br>Escape<br>Escape<br>Escape<br>Binding

								Escape

Table S2. Overview of validated observed and unobserved RBD variants. Related to STAR Methods. Variant name, fraction from which it was retrieved (observed label), Predicted label (majority of consensus), sub-library to which it belongs, Levenshtein distance from Wu-Hu-1 sequence, consensus of models and predicted label of each model for low agreement predicted variants.

Figure	Comparison	Significance
2F	No RBD vs. Wuhan	****
2F	No RBD vs. Observed Binder	****
2F	No RBD vs. Observed Escape	****
2F	Observed Escape vs. Observed Binder	****
2F	Observed Escape vs. Wuhan	****
2F	Observed Binder vs. Wuhan	ns
4C	No RBD vs. Full agreement predicted escape	****
4C	No RBD vs. Low agreement predicted escape	****
4C	No RBD vs. Low agreement predicted binder	****
4C	No RBD vs. Full agreement predicted binder	****
4C	No RBD vs. Wuhan	****
4C	Full agreement predicted escape vs. Low agreement predicted escape	****
4C	Full agreement predicted escape vs. Low agreement predicted binder	****
4C	Full agreement predicted escape vs. Full agreement predicted binder	****
4C	Full agreement predicted escape vs. Wuhan	****
4C	Low agreement predicted escape vs. Low agreement predicted binder	****
4C	Low agreement predicted escape vs. Full agreement predicted binder	****
4C	Low agreement predicted escape vs. Wuhan	****
4C	Low agreement predicted binder vs. Full agreement predicted binder	*
4C	Low agreement predicted binder vs. Wuhan	ns
4C	Full agreement predicted binder vs. Wuhan	*

Table S3. Significance comparison of validated RBD variants. **Related to Figure 2 and 4.** Full pairwise statistics performed across groups of validated RBD variants. An unpaired two-tailed Student's t test was used for all comparisons. ∗p<0.05, ∗∗p<0.01, ∗∗∗p<0.001, ∗∗∗∗p<0.0001, not significant (ns).

Model	Tuning parameters	Best parameters
Logistic Regression	max_iter: [1000, 2000, 3000, 4000]<br>C: [1, 10, 50]<br>Solver: [lbfgs, saga]	max_iter: 1000<br>C: 10<br>Solver: lbfgs
Random Forest	n_estimators: [400, 500, 550]<br>min_samples_split: [2, 5]<br>min_samples_leaf: [1, 2]<br>max_depth: [100, 150 ,200]<br>max_features: [sqrt, None]	n_estimators: 500<br>min_samples_split: 2<br>min_samples_leaf: 1<br>max_depth: 200<br>max_features: sqrt

RNN	batch_size: [16, 32]<br>drop_out: [0.1, 0.2]<br>optimizer: [adam, SGD]<br>units: [40,80]<br>learn_rate: [5e-4, 1e-4, 1e-3]	batch_size: 32<br>drop_out:.2<br>optimizer: adam<br>units: 80<br>learn_rate: 1e-3<br>max epochs: 250 with early stopping<br>patience: 10
Transformer	Tuning was performed based on the default PLMFit parameters:<br><br>Learning rate: [1e-2 to 1e-6]<br>Batch size: [16 to 512]<br>Weight decay: [1e-4 to 1e-6]	Best parameters were chosen for each run based on smallest validation loss, according to the default PLMfit fine tuning parameters.

Table S4. Key metrics for Logistic regression, Random forest and RNN models. Related to Star Methods.
Parameter tuning for the machine learning models was performed using a grid search approach, using custom scripts and optimizing model performance based on MCC.
